# Antigenic mapping reveals sites of vulnerability on α-HCoV spike protein

**DOI:** 10.1038/s42003-022-04160-8

**Published:** 2022-11-04

**Authors:** Jiangchao Xiang, Jie Su, Qiaoshuai Lan, Wenwen Zhao, Yu Zhou, Youwei Xu, Jun Niu, Shuai Xia, Qilian Qi, Sachdev Sidhu, Lu Lu, Shane Miersch, Bei Yang

**Affiliations:** 1 School of Life Science and Technology and Shanghai Institute for Advanced Immunochemical Studies, ShanghaiTech University, 393 Middle Huaxia Road, 201210 Shanghai, China; 2 CAS Center for Excellence in Molecular Cell Science, Shanghai Institute of Biochemistry and Cell Biology, Chinese Academy of Sciences, 320 Yueyang Road, 200031 Shanghai, China; 3 University of Chinese Academy of Sciences, 19 Yuquan Road, 100049 Beijing, China; 4 Key Laboratory of Medical Molecular Virology (MOE/NHC/CAMS), School of Basic Medical Sciences, Shanghai Institute of Infectious Disease and Biosecurity, Fudan University, 130 Dong An Road, 200032 Shanghai, China; 5 The Anvil Institute, School of Pharmacy, University of Waterloo, 151 Charles St. W, Kitchener, ON N2G 1H6 Canada; 6 Shanghai Clinical Research and Trial Center, 201210 Shanghai, China

**Keywords:** Virology, Electron microscopy, X-ray crystallography

## Abstract

Understanding the antigenic signatures of all human coronaviruses (HCoVs) Spike (S) proteins is imperative for pan-HCoV epitopes identification and broadly effective vaccine development. To depict the currently elusive antigenic signatures of α-HCoVs S proteins, we isolated a panel of antibodies against the HCoV-229E S protein and characterized their epitopes and neutralizing potential. We found that the N-terminal domain of HCoV-229E S protein is antigenically dominant wherein an antigenic supersite is present and appears conserved in HCoV-NL63, which holds potential to serve as a pan-α-HCoVs epitope. In the receptor binding domain, a neutralizing epitope is captured in the end distal to the receptor binding site, reminiscent of the locations of the SARS-CoV-2 RBD cryptic epitopes. We also identified a neutralizing antibody that recognizes the connector domain, thus representing the first S2-directed neutralizing antibody against α-HCoVs. The unraveled HCoVs S proteins antigenic similarities and variances among genera highlight the challenges faced by pan-HCoV vaccine design while supporting the feasibility of broadly effective vaccine development against a subset of HCoVs.

## Abstract (web summary)

The antigenic landscape of α-HCoVs S proteins is revealed, highlighting the challenges faced by pan-HCoV vaccine design but also revealing opportunities for development of broadly effective vaccines against a subset of HCoVs.

## Introduction

As RNA viruses, coronaviruses (CoVs) are constantly evolving and frequently jump from their natural reservoirs, such as bats, into humans^1^. Currently, seven CoVs can infect human, including HCoV-229E (229E) and HCoV-NL63 (NL63) from the α genus and HCoV-OC43 (OC43), HCoV-HKU1 (HKU1), MERS-CoV, SARS-CoV and SARS-CoV-2 from the β genus, all of which have a zoonotic origin^2^. Among these human CoVs (HCoVs), SARS-CoV, MERS-CoV, and SARS-CoV-2 spilled over into human population recently and are highly transmissible and pathogenic. Meanwhile, the other HCoVs, such as 229E, crossed the species barrier long ago, have adapted themselves to coexist with human and usually cause self-limiting respiratory infections, but can be lethal in children, seniors, and immunocompromised people^3^. Given the high probability of another CoV spillover within the next 10 to 50 years, the development of broadly effective countermeasures against CoVs is a global priority^4^. Nevertheless, despite recent advances in vaccines and therapeutics development against SARS-CoV-2, no vaccines with pan-HCoV activity are currently available.

The spike (S) proteins of CoVs mediate their host entry and is the major target of vaccine or therapeutic development against HCoVs^5^. The S protein is composed of two subunits, S1 and S2. The S1 subunit contains the N-terminal domain (NTD) and C-terminal domain (CTD), both could be engaged in host receptor recognition and viral attachment^6^. Meanwhile, the S2 subunit is a spring-loaded fusion machinery^7^. Prior to host receptor attachment, the S protein generally adopts a metastable pre-fusion conformation wherein its S1 trimer caps the trimeric S2 stalk. Upon host receptor engagement and proteolytic separation of S1 and S2, the otherwise buried fusion peptides (FPs) in S2s become exposed and insert themselves into the host membrane, which in turn triggers the rearrangement of the heptad repeats (HRs) within S2s to form the 3HR1-3HR2 six-helical bundle (6-HB), thereby bringing viral and host membranes into proximity and facilitating membrane fusion^7,8^. Of note, drastic differences exist between the pre-fusion conformations of α- and β-HCoVs S proteins, such as the different packing modes between their NTDs and CTDs^9–11^. Besides, while the CTDs from SARS-CoV, MERS-CoV and SARS-CoV-2 sample ‘up’ and ‘down’ conformations with comparable frequencies in pre-fusion states, the CTDs from α-HCoVs have only been captured in the receptor-inaccessible ‘down’ conformation^12–15^. Together, these structural differences may lead to different immunogenicity of α- and β-HCoVs S proteins.

The successive emergence of SARS-CoV and MERS-CoV in this century, and the unprecedented SARS-CoV-2 pandemic fueled the discovery of neutralizing antibodies (NAbs) against them, either from patient sera or from antibody libraries and S protein-immunized mice^16,17^. Most of these reported NAbs target their CTDs, which are also their receptor binding domains (RBDs), while the remaining few recognize the NTDs or S2 subunits, indicating an immunodominant role of RBDs in these three β-HCoVs^18,19^. Indeed, it was reported that 90% of the neutralizing activity present in COVID-19 convalescent sera is directed against SARS-CoV-2 RBD^20^. While RBD-directed NAbs generally work by directly or indirectly blocking the interaction between S proteins and host receptors, the working mechanism of NAbs directed to other regions on S protein may involve steric hinderance of host receptor binding or inhibition of pre-to-post conformational changes of S proteins^21^. Notably, NTD-directed NAbs against these three highly pathogenic β-CoVs frequently recognize a similar outward-facing, glycan-free area on their NTDs (Supplementary Fig. 4a)^22–27^, and a single ‘antigenic supersite’ is even found to be recognized by almost all SARS-CoV-2 NTD-specific NAbs^25^. Coincidentally, two recent studies on the common cold β-HCoV, OC43, also revealed the existence of two adjacent ‘antigenic supersites’ in the NTD of it S protein^28,29^. Compared to the S1 cap, the fusion apparatus S2 appears to be much more conserved across different HCoVs. Nevertheless, although the S2 base may serve as an intervention target with better cross-species potential^30^, the antigenic description of S2 subunits remains limited. Thus far, only a handful of S2-directed NAbs have been described for the aforementioned three highly pathogenic β-HCoVs^31–38^, and no such NAbs have been reported yet for the α-HCoVs.

Compared with the extensive antibody studies on β-HCoVs S proteins, the discovery and characterization of NAbs against α-HCoVs S proteins have been extremely scarce. In order to explore the feasibility of pan-HCoV vaccine development and to identify potential pan-HCoV epitopes on S proteins, it is important to understand the immunogenic variance of S proteins among different HCoVs, wherein depicting the antigenic landscapes of S proteins from α genus is currently missing. Here, we took 229E as the representative α-HCoV and aim to unravel the antigenic signatures of its S protein. We identified a panel of antibodies against the NTD, CTD (also its RBD) and S2 subunit of the 229E S protein from a phage-displayed library and characterized the neutralizing potencies of selected antibodies. Next, we utilized an integrative structural biology approach that combines hydrogen-deuterium-exchange coupled mass-spectrometry (HDX-MS)^39^, cryo-EM and X-ray crystallography to understand the binding mode of each antibody. Together, these works revealed the antigenic features of the 229E S protein and enabled us to better appreciate the distinctions as well as similarities between α- and β-HCoVs S proteins, which not only highlights the challenges faced by pan-HCoV vaccine design but also reveals opportunities for the development of broadly effective vaccines against a subset of HCoVs.

## Results

### Selection and validation of antibodies against 229E S trimer

**Fig. 1:**
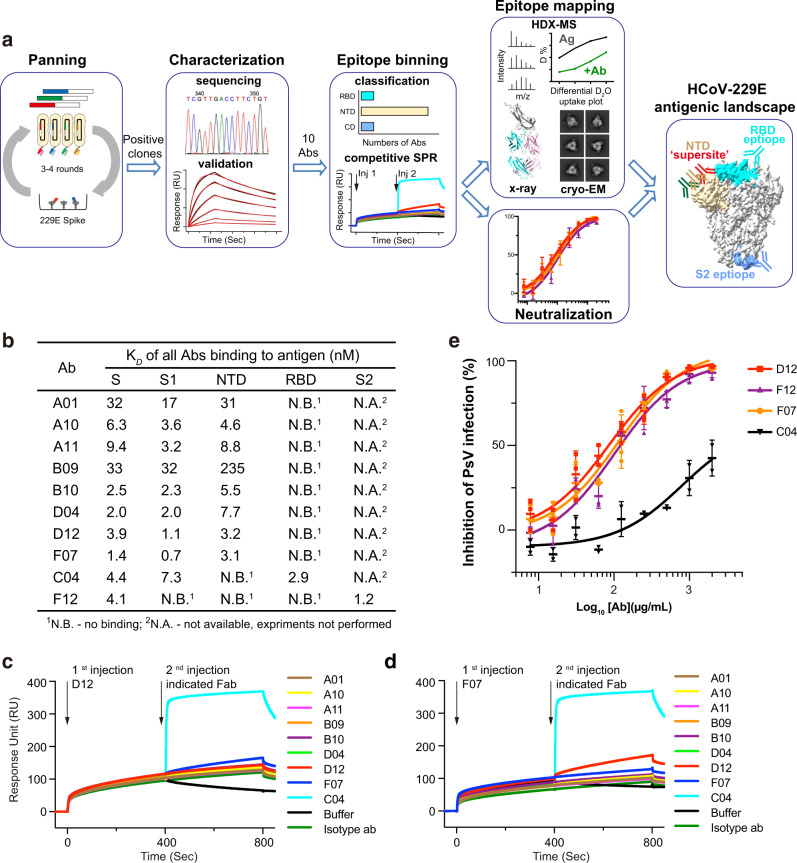
Selection, classification, and neutralizing potency characterization of antibodies against the 229E S trimer. **a** Schematic overview illustrating the antigenic landscape mapping process of 229E S trimer. **b** Interaction kinetics between each Fab and 229E S trimer, S1 or S2 subunit, NTD and RBD were characterized by SPR. The results indicate that C04 is directed against 229E RBD, F12 is directed against the S2 subunit, while all the other Fabs specifically interact with 229E NTD. **c**, **d** Competitive SPR results show that the eight NTD-directed Fabs all compete for a similar binding interface on 229E NTD. 229E S1 was immobilized onto the sensor, then blocked with saturating Fab D12 (**c**) or F07 (**d**), followed by a second injection of buffer only or the indicated Fabs. Fab C04 served as positive controls. **e** Neutralization profiles of IgG D12 (red), F12 (purple), F07 (orange) and C04 (black) against 229E PsV. Neutralizing activities are represented as individual data points and mean ± SD (*n* = 3 biologically independent experiments). Mean ± SD is indicated with . Individual data points are shown as filled red squares (D12), purple triangles (F12), orange circles (F07) and black triangles (C04), respectively.

We used a phage-displayed library (library F) of synthetic, human antigen-binding fragments (Fabs) to select for antibodies that could recognize 229E S trimer (Fig. 1a, Supplementary Fig. 1a–c). Library F has a theoretical diversity of 3 × 10^10^, and has succeeded in selections against numerous proteins, including multiple therapeutically important targets, such as IL-18, integrin, and Frizzled receptors^40–43^. Four consecutive enrichment steps of panning were performed against 229E S trimer, and single positive clones that bound to 229E S trimer but not to negative control proteins in ELISA were subjected to DNA sequencing. In total, thirteen Fabs with unique complementary-determining region (CDR) sequences were identified (Supplementary Fig. 1d). Recombinant Fabs were then purified and tested for their binding to 229E S trimer via surface plasmon resonance (SPR). Ten of the thirteen Fabs bound 229E S trimer with equilibrium disassociation constant (*K*_D_) ranging from 1–50 nM (Fig. 1b, Supplementary Fig. 1e). Three Fabs B03, B06, and E12 exhibited little or no binding to 229E S protein and thus were excluded from subsequent investigations (Supplementary Fig. 1e).

### Antibody classification and downselection

Next, we expressed and purified 229E NTD, RBD, S1, and S2 subunits (Supplementary Fig. 2a, b) and evaluated their binding to the ten validated Fabs individually (Fig. 1b, Supplementary Fig. 2c–f). Nine of the ten Fabs bound the S1 subunit efficiently, with *K*_D_ values comparable to their affinities for intact 229E S trimer (Fig. 1b, Supplementary Fig. 2c). Meanwhile, Fab F12 showed no binding at all to either S1, NTD or RBD although it bound intact S trimer tightly (Fig. 1b, Supplementary Fig. 2c, d), suggesting that it may bind to S2 subunit or a structural epitope that only exists in trimeric S. Indeed, F12 bound S2 and S trimer with comparable K_*D*_ values (1.2 and 4.1 nM) (Fig. 1b, Supplementary Fig. 1e, Supplementary Fig. 2d), confirming that the epitope of F12 is confined to the S2 subunit. Among the nine Fabs that bound the S1 subunit, only Fab C04 recognized RBD while the other eight antibodies all bound NTD (Fig. 1b, Supplementary Fig. 2e, f). Such phenomenon implies that the NTD of 229E is more antigenic than its RBD, which is in sharp contrast to previous observations on the three highly pathogenic β-HCoVs wherein their RBDs rather than NTDs are immunodominant^18,19^.

Thus far, ten 229E S-reactive Fabs could be classified into 3 major groups, each recognizing the NTD, RBD or S2 subunit of 229E S (Fig. 1b). Compared to group 2 and group 3 which include C04 and F12, respectively, the NTD-recognizing group 1 is a large group comprising eight different Fabs. To understand if these Fabs bind to similar or distinct epitopes on NTD, we then performed competitive SPR to check for their reciprocal binding to 229E S1 subunit. Considering that antibody pairs targeting similar epitopes can manifest false mutual binding when the affinity of the second antibody is much higher than that of the first one, we thus flowed the strongest NTD binder D12 (or F07) first to saturate the immobilized S1 proteins before challenging with each of the other NTD Fabs (Fig. 1c, d). Second injection of RBD-binding C04 was used as a positive control and indeed induced a significant shift in the interference pattern, while second injection of D12 (or F07) was taken as control for effective blockade (Fig. 1c, d). Second injection of any other NTD Fabs hardly elicited wavelength shift, indicating that their binding was blocked in the prior presence of D12 (or F07) (Fig. 1c, d). Moreover, concurrent binding was not observed between F07 and D12 either (Fig. 1c, d). Together, these results implies that the eight NTD-directed Fabs, A01, A10, A11, B09, B10, D04, D12 and F07, all compete for similar binding sites on NTD.

### Epitopes and neutralizing potencies of dominant NTD-directed antibodies

**Fig. 2:**
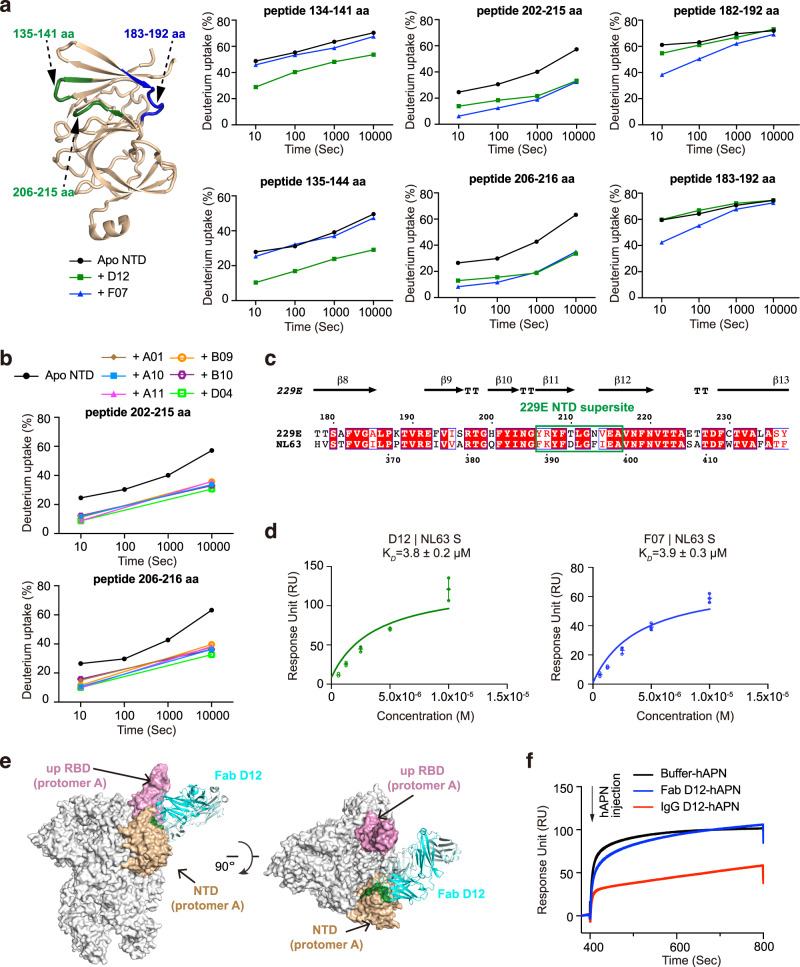
NTD-directed antibodies all target a same antigenic supersite on 229E. **a** Antibody-dependent amide hydrogen protection indicates the potential interacting regions of D12 and F07 on 229E NTD. 229E NTD segments that manifested considerable HDX decrease at all measured timepoints in the presence of Fab D12 or F07 are highlighted on the cartoon representation of NTD and labeled. Deuterium uptake plots of these segments in the absence (black) and presence of Fab D12 (dark green) or F07 (blue) are plotted as percent deuterium uptake versus time on a logarithmic scale to illustrate their HDX kinetics. **b** Deuterium uptake plots show that considerable HDX decreases were observed for NTD peptides 202–215 aa and 206–216 aa at 10 s and 10,000 s in the presence of all other 229E NTD-directed antibodies, indicating that these antibodies also target this region on 229E NTD. **c** Sequence alignment of 229E and NL63 NTDs. The antigenic supersite in 229E NTD is marked by green rectangle. **d** Equilibrium analysis plots depicting the interactions between immobilized NL63 S protein and Fabs D12 (left) and F07 (right). The K_*D*_ values are shown as individual data points and mean ± SD (*n* = 3 biologically independent experiments). Mean ± SD is indicated with  and individual data points are represented with open green (D12) or blue (F07) circles. **e** Model of Fab D12 binding to 229E S trimer in one-RBD-up configuration. Fab D12 is depicted as ribbons (light and heavy chains in different shades of blue). 229E S trimer is shown as gray surface representations, with its RBD in ‘up’ configuration colored pink and the intra-subunit NTD colored wheat. NTD region 206–215 aa is colored dark green to highlight the consensus epitope targeted by D12, F07 and all the other NTD-directed antibodies. **f** Pre-binding of D12 in IgG but not Fab format interferes with the binding of hAPN to the 229E S trimer. Immobilized 229E S trimer was saturated with D12 in either Fab or IgG format before injection of recombinant hAPN protein (injection point indicated with arrow). All SPR experiments were independently performed at least twice and representative profiles from one experiment are shown.

Previous studies of the β-HCoVs S proteins revealed the existence of ‘antigenic supersites’ in the NTDs of SARS-CoV-2 and OC43^25,28,29^. To understand if the NTDs of α-CoVs exhibit similar antigenic features, we set out to map the epitopes of the eight NTD-directed Fabs and assess their neutralizing potential. We first utilized HDX-MS^39^ to fine map the epitope of D12 on the NTD of 229E (Fig. 2a, Supplementary Figs. 3a, 7a). Compared to NTD alone, peptides 134–141 aa, 135–144 aa, 202–215 aa and 206–216 aa manifested markedly decreased HDX at all measured timepoints in the presence of Fab D12, suggesting that NTD regions encompassing 135–141 aa and 206–215 aa are likely involved in D12 interactions (Fig. 2a, Supplementary Fig. 3a). Next, epitope mapping was performed for all the other NTD antibodies using HDX-MS (Supplementary Fig. 3). Of note, some of the antibodies manifested similar HDX protection profiles as seen with D12 (Supplementary Fig. 3a), while others, exemplified by F07, exhibited profound HDX protection in regions encompassing 183–192 aa and 206–215 aa instead (Fig. 2a, Supplementary Fig. 3b). As 135–141 aa, 183–192 aa and 206–215 aa are spatially close to each other (Fig. 2a) and all antibodies manifested strongest HDX protection in 206–215 aa (Fig. 2a, b; Supplementary Fig. 3), the HDX-MS results thus suggest that the NTD loop encompassing 206–215 aa likely plays determinant roles in engaging all NTD antibodies. These antibodies, divided into two sub-groups and represented by F07 and D12 respectively, may approach 206–215 aa from slightly different angles, thereby leading to different protection profiles on peripheral regions like 135–141 aa and 183–192 aa. Consistent with the HDX-MS results, Ala-mutations of single residues within 206–215 aa caused up to 96% decrease in antibody binding, and the tetra-mutant (NTD^T210A/L211A/N213A/V214A^) barely bound D12 or F07 anymore (Supplementary Table 1). Hence, consistent with the results from competitive SPR, the HDX-MS data also indicates that the epitopes of the eight NTD-directed Fabs are highly similar and represent an ‘antigenic supersite’ in the 229E NTD (Supplementary Fig. 4b, close-up view).

Next, we asked whether this antigenic supersite, 206–215 aa, is preserved in NL63, another α-HCoV. Notably, curated prediction of the B cell epitopes on NL63 NTD indicates the presence of a strong B cell epitope at the same position (Supplementary Fig. 4d, far right panel, 393–397 aa). Moreover, this NTD epitope manifested ~80% sequence similarity among the various isolates of α-HCoVs (Fig. 2c, boxed region and Supplementary Fig. 4e). Indeed, Fabs D12 and F07 could cross-react with NL63 S protein (Fig. 2d and Supplementary Fig. 4f) (albeit weakly), most likely driven by their recognition of the similar region in NL63 NTD (Fig. 2c, boxed region). The above results collectively argue that, akin to previous observations in β-HCoVs, ‘antigenic supersites’ also exist in the NTDs of α-HCoVs, although the locations of such ‘antigenic supersites’ are distinct between α-HCoVs and β-HCoVs (Supplementary Fig. 4d).

Unlike the outward-facing antigenic supersites in β-HCoVs NTDs (Supplementary Fig. 4a)^22–29^, the identified antigenic supersite in the NTD of 229E appears to be partially buried in the all-RBD-down conformation of the 229E S trimer (Supplementary Fig. 4b), although it would become fully accessible when the intra-subunit RBD is in the ‘up’ conformation (Supplementary Fig. 4c). We then docked D12 onto the one-RBD-up model of 229E S trimer, using 206–215 aa of NTD as interface constraints (Fig. 2e). Based on the docking model, the binding of D12, in its IgG format, would hinder the interaction between 229E RBD and its host receptor human aminopeptidase N (hAPN)^44,45^. Indeed, we found that pre-incubation of 229E S with IgG D12 reduced the binding of hAPN (Fig. 2f) and that IgG D12 inhibited the entry of 229E pseudovirus (PsV) with IC_50_ at 86 μg/mL (Fig. 1e). Likewise, IgG F07 also inhibited the entry of 229E PsV with IC_50_ at 115 μg/mL (Fig. 1e).

Collectively, these results indicate that all the 229E NTD-directed antibodies recognize a similar neutralizing epitope ranging 206–215 aa on 229E NTD. Given the high sequence conservation of this epitope across various isolates of α-HCoVs (Supplementary Fig. 4e), it may even represent a hotspot of antigenic vulnerability on all α-HCoVs NTDs.

### Epitope and neutralizing potency of the first α-HCoV S2-directed antibody F12

**Fig. 3:**
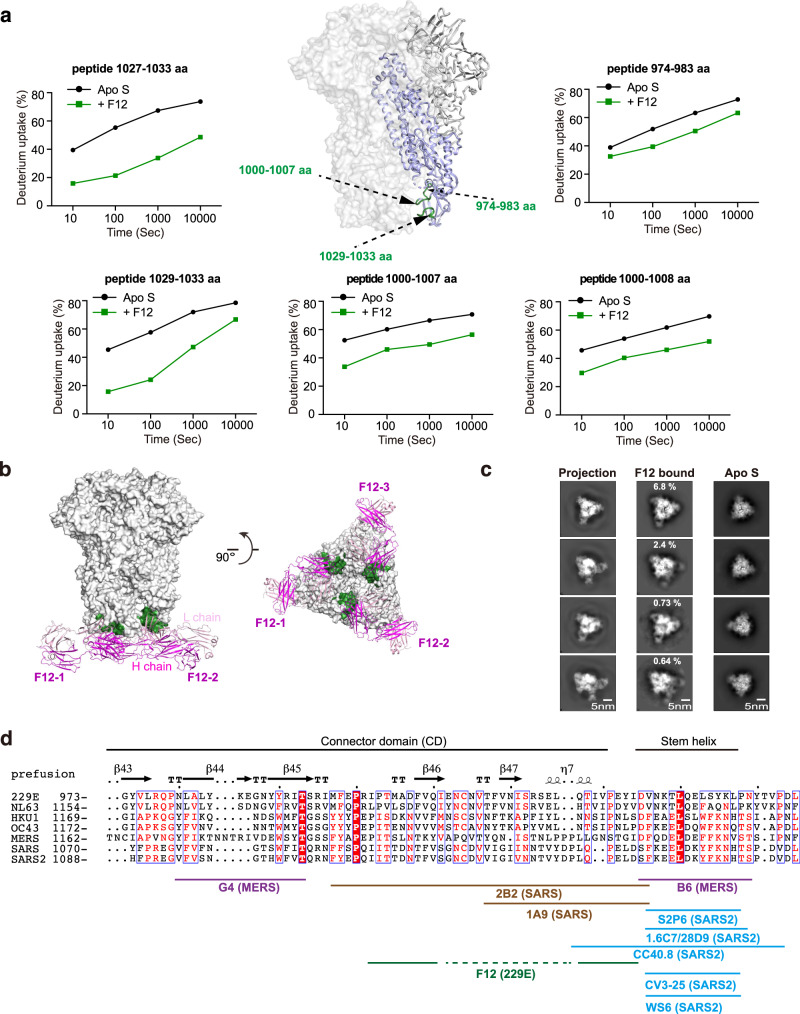
F12 binds the CD of the 229E S protein and lacks cross-neutralizing activity to NL63. **a** F12-dependent amide hydrogen protection indicates its potential interacting regions in 229E S2. S2 segments that manifested considerable HDX decrease at all measured timepoints in the presence of Fab F12 are highlighted with dark green on the cartoon representation of one 229E S protomer (S2 colored purple) in the context of 229E S trimer (light gray surface presentation). Deuterium uptake plots of these segments in the absence (black) and presence (dark green) of Fab F12 are plotted as percent deuterium uptake versus time on a logarithmic scale to illustrate their HDX kinetics. **b** Model of Fab F12 binding to 229E S trimer in the all-RBD-down state. Fab F12 is depicted as ribbons (light and heavy chains in different shades of magenta). The authentic epitope of F12 as double-defined by HDX-MS results and mutagenesis verification is highlighted with dark green on the gray surface representation of 229E S trimer. **c** To examine the correspondence between cryo-EM 2D class averages and the model of F12-bound 229E S trimer, representative 2D projections of the model (left panels) is compared to reference-free averages of F12-bound 229E S trimer (middle panels) or 229E S trimer alone (right panels). **d** Sequence alignment of CD and stem helix region within the S2 subunits of α- and β-HCoVs. The structural epitope of F12 and the epitopes of other previously reported S2-directed antibodies are indicated with solid lines under the alignment.

The stronger potential of S2 subunit over S1 subunit to serve as pan-HCoV intervention target has spurred enthusiasm to search for S2-directed antibodies^46,47^. Although a few S2-directed antibodies have been reported for SARS-CoV, SARS-CoV-2 and MERS-CoV and showed limited broadness among them, no such antibody has been reported yet for α-HCoVs until the identification of F12 in this study^31–38^ (Fig. 1b). We thus went on to fine map the epitope of F12 on intact 229E S trimer and to characterize its breadth within genus. In the presence of F12, decreased HDX was observed for peptides 974–983 aa, 1000–1007 aa, 1000–1008 aa, 1027–1033 aa and 1029–1033 aa in 229E S2 (Fig. 3a, Supplementary Figs. 5a, 7b), indicating a high probability for residues in these regions to interact with F12 directly or indirectly. The HDX protection effect was most profound in regions ranging 1027–1033 aa, followed by 1000–1007 aa, and was moderate in 974–983 aa (Fig. 3a; Supplementary Fig. 5a). Such observation, together with the fact that 1000–1007 aa and 1029–1033 aa are conformationally adjacent to each other (Fig. 3a), suggest that these two regions likely constitute the authentic epitope for antibody F12, while the HDX decrease in spatially more distant 974–983 aa may originate from indirect stabilizing effects. Consistent with such notion, single residue mutagenesis in either 1000–1007 aa or 1029–1033 aa completely prevented the binding of F12 (Supplementary Table 2).

Using these findings as constraints, we then went on docking F12 onto the pre-fusion conformation of 229E S trimer (Fig. 3b)^10^. To further confirm the docking results, we also collected cryo-EM data on F12-bound 229E S trimer. Although a high-resolution model of this complex was not obtained due to strong orientation preference, we were able to obtain high quality reference-free class averages of some views (Fig. 3c). Comparing the reference-free class averages of F12-bound and F12-free 229E S trimer, stoichiometric binding of F12 could be clearly seen (Fig. 3c, compare the right two panels). Moreover, the reference-free class averages of F12-bound 229E S trimer matched very well to the 2D projections of the docking model (Fig. 3c, compare the left two panels), further corroborating the docking model. Next, we tested the neutralizing potency of F12. IgG F12 did inhibit the entry of 229E PsV with IC_50_ at 101 μg/mL (Fig. 1e), possibly by initiating premature conformational change of S proteins or by blocking the formation of the 6-HB, just like other S2-binding NAbs do^35,38^. Collectively, these results indicate that F12 recognizes a structural epitope in the connector domain (CD) of 229E S2 subunit and is indeed neutralizing (Fig. 3d).

Previously, a few S2-directed NAbs have been reported^31–38^. Of the two NAbs that target MERS-CoV S2, G4 binds to a variable glycosylated loop within the CD and lacks cross-reactivity, while B6 recognizes the relatively conserved stem helix region immediately before the HR2 and has been found to cross neutralize MERS-CoV and OC43 (Fig. 3d)^35,38^. Meanwhile, 2B2 and 1A9 recognize similar regions within the CD of SARS-CoV and the latter manifests limited cross-reactivity to SARS-CoV-2 (Fig. 3d)^37,48^. For SARS-CoV-2, the epitopes of all the S2-directed NAbs cluster within the stem helix region and one of these NAbs, S2P6, has been found to harness broad neutralizing activity against nearly all β-HCoVs^31–36^ (Fig. 3d). Taken together, these findings indicate that the stem helix region within S2 seems to be an epitope with better pan-HCoV potential than the CD region. In further support of this, we found that CD-targeting F12 lacks cross-reactivity even to NL63 in the same genus (Supplementary Fig. 5b).

### Neutralizing epitopes in the distal end of 229E RBD

**Fig. 4:**
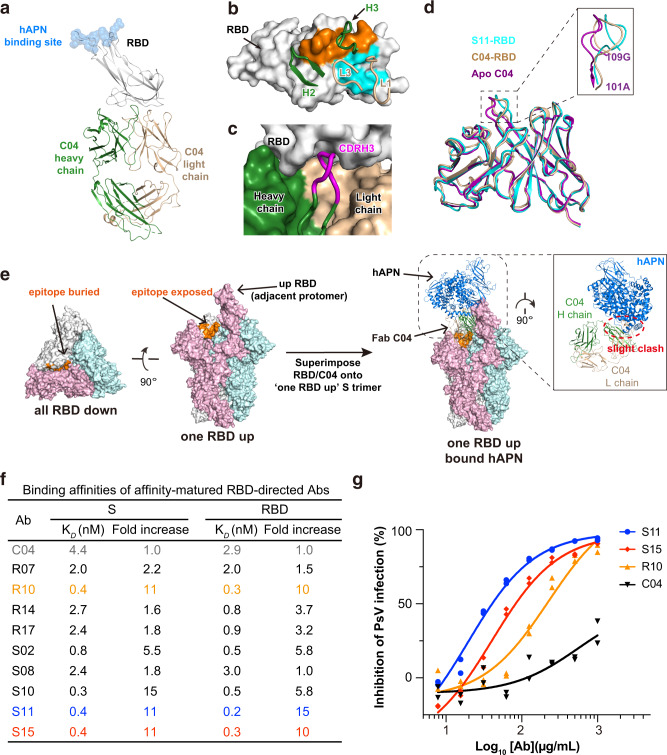
C04 binds to the distal end of 229E RBD and neutralizes 229E PsV after affinity maturation. **a** Crystal structure of Fab C04 in complex with 229E RBD in cartoon representations. The heavy and light chain of C04 are colored dark green and wheat respectively, while 229E RBD are colored gray. The binding site of host receptor hAPN is depicted with blue spheres. **b** Contact residues are colored orange (heavy chain) and blue (light chain) on surface representation of 229E RBD. CDR loops engaging 229E RBD are shown and labeled. **c** The heavy (dark green) and light chain (wheat) of C04 and the RBD (gray) are shown as surface representations. CDRH3 (magenta) is depicted in cartoon to illustrate its neat fitting into the surface pocket on 229E RBD. **d** Structural superimposition of free C04 (purple), RBD-bound C04 (wheat) and RBD-bound S11(blue) illustrating the conformational change of CDRH3 upon binding RBD. All structures are shown as cartoon representations and the inset is the close-up view of CDRH3 region. **e** Left: The epitope of C04 appears buried in all-RBD-down conformation but becomes fully exposed in one-RBD-up conformation. Right: Binding of Fab C04 would slightly interfere with the binding of hAPN to neighboring ‘up’ protomer (colored pink, anti-clockwise). Structure of Fab C04-bound 229E S trimer was modeled by superimposing the structure of RBD-C04 onto the one-RBD-up 229E S trimer. Next, this structure was aligned with hAPN-229E RBD complex based on the 229E RBD in ‘up’ position. **f** Binding affinities of parental C04 and affinity-matured antibodies against 229E S trimer or RBD were characterized by SPR. Of the affinity-matured antibodies, antibody S11 (blue) and S15 (red) exhibited up to 11-fold increase in their affinity for 229E S trimer and RBD. **g** Neutralization profiles for IgG S11 (blue), S15 (red) and R10 (orange) are compared to that of parental IgG C04 (black) against 229E PsV. Individual data points are shown as filled blue circles (S11), red diamonds (S15), orange triangles (R10) and black triangles (C04) respectively.

In the case of SARS-CoV-2, it is noticed that the most potent NAbs usually recognize epitopes within RBD, followed in potency by antibodies against its NTD and S2 subunit^20,49,50^. Contrary to this observation, the RBD-directed C04 appears to be the least neutralizing in all identified antibodies against 229E S, with IC_50_ > 500 μg/mL (Fig. 1e). To understand if its low neutralizing activity correlates with its mode of action, we then determined the crystal structure of Fab C04 alone and C04 in complex with 229E RBD (Fig. 4a, Supplementary Fig. 6a and Table 1). C04 buried 525 Å^2^ surface area on the RBD with its CDRL1 and CDRL3, and 462 Å^2^ with its CDRH2 and CDRH3 (Fig. 4b). Among these CDR loops, CDRH3 is of particular interest as it contains as many as five Gly residues within the CDRH3 loop (Supplementary Fig. 1d), which likely endows it with great structural plasticity to neatly fit itself into a pocket on RBD surface (Fig. 4c), through largely backbone driven interactions (Supplementary Fig. 6g). The structural plasticity of CDRH3 is also well demonstrated by its large conformational change upon binding RBD (Fig. 4d). While the RMSD of CDRH3 reached 1.51 Å upon RBD binding, the average RMSD of CDRL1, CDRL3 and CDRH2 was only 0.66 Å.

**Table 1:** X-ray data collection and refinement statistics.

	C04 Fab	RBD-C04	RBD-S11
PDB ID	7VMZ	7VN9	7VNG
*Data collection*			
Space group	*P* 6_2_	*P* 6_2_22	*C* 222
Cell dimensions			
* a, b, c* (Å)	148.2, 148.2, 53.4	183.7, 183.7, 255.9	113.6, 174.7, 97.4
* α, β, γ* (°)	90°, 90°, 120°	90°, 90°, 120°	90°, 90°, 90°
Resolution range (Å)^*^	50.0–2.9 (3.0–2.9)	50.0–4.6 (4.8–4.6)	49.1–3.8 (4.3–3.8)
No. of observed reflections	297,153	254,462	122,460
No. of unique reflections	15,842	14,882	9,887
Completeness (%)^*^	99.9 (100)	100 (100)	100 (100)
Redundancy^*^	18.7 (13.2)	17.1 (11.7)	12.4 (12.8)
*R*_*meas*_ (%)^*^	28.9 (>100)	37.2 (>100)	20.0 (>100)
*R*_*pim*_ (%) ^*^	6.5 (32.3)	8.9 (47.3)	5.7 (47.5)
<*I/σ(I)*>^*^	16.4 (2.3)	6.5 (1.2)	7.8 (1.8)
CC_1/2_(%)^*,a^	97.6 (87.0)	99.6 (60.6)	99.8 (87.3)
Wilson B (Å^2^)	65.9	173.4	147.1
*Refinement*			
Resolution (Å)^*^	35.6–2.9 (3.0–2.9)	36.9–4.6 (4.8–4.6)	49.1–3.8 (3.9–3.8)
Reflections used in refinement^*^	15,820 (1,568)	14,756 (1,417)	9,857 (967)
Reflections used for *R*_free_^*^	890 (75)	748 (78)	984 (97)
*R*_*work*_ (%)^*^	22.3 (28.9)	27.1 (32.3)	27.3 (31.2)
*R*_*free*_ (%)^*^	26.4 (36.3)	31.3 (37.7)	29.8 (35.9)
No. atoms	3,272	8,509	3,957
Protein	3,272	8,389	3,929
Glycans	0	120	28
*B*-factors (Å^2^)	67.5	193.1	181.3
Protein	67.5	193.1	181.2
Glycans	0	196.4	188.2
R.m.s. deviations			
Bond length (Å)	0.004	0.006	0.003
Bond angle (°)	0.7	1.1	0.7

^*^ Values in parentheses are for highest-resolution shell.

^a^ $${{CC}}_{\frac{1}{2}}=\frac{\sum (x-\left\langle x\right\rangle )(y-\left\langle y\right\rangle )}{{\left[\sum {(x-\left\langle x\right\rangle )}^{2}\sum {(y-\left\langle y\right\rangle )}^{2}\right]}^{\frac{1}{2}}}$$.

Notably, the epitope of C04 is distal to the hAPN receptor binding site on 229E RBD (Fig. 4a), somewhat analogous to the binding mode of several SARS-CoV-2 NAbs including CR3022, EY6A, S304 and 553-49, which also target the ‘cryptic’ epitopes in the distal end of SARS-CoV-2 RBD^20,51–53^. Akin to these SARS-CoV-2 NAbs, the epitope of C04 also appears buried in the all-RBD-down conformation of 229E S trimer, yet would become fully accessible when its anti-clockwise neighboring RBD is in the ‘up’ position (Fig. 4e, left panel). Structural alignment of the C04-229E RBD complex with the one-RBD-up S trimer further indicates that binding of Fab C04 would slightly interfere with the binding of hAPN to the neighboring ‘up’ protomer (Fig. 4e, right panel). Consistently, pre-incubation of 229E S with C04 in either Fab or IgG format indeed reduced the binding of hAPN (Supplementary Fig. 6b), although to an extent less than that induced by IgG D12 pre-incubation (Fig. 2f). Of note, binding of CR3022 or 553-49 has been shown to induce the destruction of SARS-CoV-2 S protein^53–55^. As C04 also targets the ‘cryptic’ surface on 229E S protein, we wondered if the binding of C04 would induce the disassembly of 229E S and thus visualized 229E S under negative-staining EM either in the absence or presence of C04 (Supplementary Fig. 6c). In the presence of C04, a good portion of the 229E S trimers disassembled into C04-bound 229E S protomer (Supplementary Fig. 6c, right) by the end of the 1 h incubation while most of the 229E S trimers remained intact in the absence of C04 (Supplementary Fig. 6c, left). Therefore, besides blocking the binding of APN through steric hinderance, C04 might also neutralize through inducing the disassembly of 229E S protein.

The position of C04 epitope explains its relative low neutralizing activity. In the case of distal-end binding antibodies like CR3022, neutralizing activity is highly correlated with their affinity for SARS-CoV-2 RBD^56^. Given that C04 was selected from a naïve synthetic library, it obviously lacks the opportunity to improve its affinity through somatic hypermutation. To enhance the neutralizing potency of C04, we thus performed in vitro affinity maturation using a soft-randomized library based on the parental clone and obtained a subset of positive clones which exhibit higher affinities for both 229E RBD and S trimer (Fig. 4f, Supplementary Fig. 6d, e). Of these affinity-matured antibodies, antibody S11 and S15 exhibited up to an 11-fold increase in their affinities for 229E RBD and S trimer (Fig. 4f), largely due to their much slower disassociation from targets (Supplementary Fig. 6d, e). Accordingly, IgG S11 and S15 manifested significantly enhanced neutralizing activity against 229E PsV infections as compared to IgG C04, with IC_50_ values at 19 and 40 μg/mL respectively (Fig. 4g). We also went further to characterize the structure of S11-RBD complex and found that the binding mode of S11 is the same to that of C04 (Supplementary Fig. 6f), with an overall RMSD of 0.69 Å. The major difference between S11 and C04 lies in their unique G-rich CDRH3 loops, wherein CDRH3 residues A^109^S^110^V^111^ matured into I^109^T^110^F^111^ in S11, thereby enabling additional sidechain-to-sidechain hydrophobic packing with P393 and W419 of RBD (Supplementary Fig. 6g). Hence, analogous to the ‘cryptic’ epitopes in the distal end of SARS-CoV-2 RBD, a neutralizing epitope is also located in the distal end of 229E RBD.

## Discussion

To date, an antigenic understanding of the α-HCoVs S proteins has been lacking. In this study, we took 229E as the representative α-HCoV and conducted antigenicity mapping of its S protein. Due to our lack of access to convalescent sera samples from α-HCoVs infected individuals, we opted to leverage a highly validated, synthetic Fab library to select antibodies that recognize trimeric 229E S (Fig. 1a). Of the ten NAbs identified, eight recognize the NTD, suggesting that the NTD of 229E is antigenically dominant (Fig. 1b). Such observation is in sharp contrast to previous studies on SARS-CoV, MERS-CoV and SARS-CoV-2 S proteins, wherein their RBDs instead of NTDs are most frequently targeted by NAbs^18,19^. Consistently, recent finding from Shi *et al*. also found that the ability of 229E RBD to induce neutralizing potency in mouse sera is significantly lower than that of intact 229E S trimer or 229E S1^57^.

We also delineated the structural epitopes of selected NAbs combining HDX-MS, cryo-EM and crystallography. Prior to this study, only a few S2-directed antibodies have been discovered against the three highly pathogenic β-HCoVs^31–38^, which target either the CD or the stem helix region that locates immediately upstream of the HR2 motif (Fig. 3d). The antibody F12 from this study represents the first identified NAb against the S2 subunit of an α-HCoV and it recognizes structural epitopes within the CD (Fig. 3). Of note, F12 lacks cross-reactivity even to NL63, a close neighbor to 229E in the same genus (Supplementary Fig. 5b). Likewise, the G4 antibody that targets the CD of MERS-CoV also lacks cross-reactivity, and SARS-CoV CD-targeting antibodies 2B2 and 1A9 only manifest limited cross-reactivity to SARS-CoV-2^37,38,48^. In contrast, antibodies B6 and S2P6 which target the stem helix region of MERS-CoV and SARS-CoV-2 respectively appear to harbor much broader cross-reactivity. While B6 cross-neutralizes MERS-CoV and OC43, two β-HCoVs from different subgenera, S2P6 provides broad neutralizing activity against nearly all β-HCoVs^35,36^. Collectively, these findings indicate that the stem helix region within S2 seems to be a better target than the CD if broadly effective antibodies are to be elicited. During the submission of this work, three independent studies have identified the FP as another potential pan-HCoV intervention target within the S2 subunit^58–60^. Nevertheless, it is worth noting that, either the stem helix region or the FP epitope is partially buried in the pre-fusion state of S trimers and thus may not be readily accessible for host immune recognition^36,58–60^. The inferior antigenicity of the stem helix region is also exemplified by the fact that we did not obtain any antibody targeting this region of 229E S in this study, while CD-binding F12 was identified (Fig. 3). Hence, to fully explore the potential of the stem helix or the FP epitopes in broadly effective vaccine design, immunofocusing approaches are necessary to concentrate the host response towards these two epitopes, such as masking the less broad yet more antigenic epitopes nearby (e.g., the CD epitope) and enhancing their accessibility through structure-guided immunogen presenting^61^.

Besides the neutralizing epitope within the S2, neutralizing epitope was also captured in the end distal to the hAPN binding site on 229E RBD (Fig. 4), reminiscent of the cryptic epitopes recognized by NAbs CR3022, EY6A, S304 and 553-49 in the distal end of SARS-CoV-2 RBD^20,51–53^. Notably, binding of CR3022 or 553-49 has been found to induce the disassembly of SARS-CoV-2 S protein^53–55^. Akin to them, binding of C04 to the ‘cryptic’ epitope in 229E RBD also induces the destruction of 229E S trimer, suggesting that induced disassembly of S trimer may be a common mechanism for NAbs that target the ‘cryptic epitopes’ buried in the distal end of CoVs RBD. In the NTD of 229E, a region comprising 206–215 aa is recognized by all eight 229E NTD-directed antibodies (Figs. 1c, d, 2a, b and Supplementary Fig. 3), indicating the high antigenicity of this region and reminding us of the previously reported ‘antigenic supersites’ in SARS-CoV-2 and OC43 NTDs (Supplementary Fig. 4a)^25–29^. Notably, this 229E NTD ‘antigenic supersite’ seems to be conserved within α-HCoV (Fig. 2c and Supplementary Fig. 4e), and two 229E antibodies targeting this supersite are cross-reactive to NL63 (Fig. 2d and Supplementary Fig. 4f), the other α-HCoV. Together, these facts suggest that this NTD supersite holds potential to serve as a broadly effective epitope within α-HCoVs.

To summarize, here we took 229E as the representative α-HCoV and defined the antigenic landscape of its S protein. While these findings highlight the antigenic variance of S protein across genera and the challenges faced by pan-HCoV vaccine design, they also revealed some shared features, which may provide information for development of broadly effective vaccines against at least a subset of HCoVs. We anticipate that our work would serve as a starting point to comprehensively understand the similarities and distinctions among all circulating HCoVs, *esp*. between α-HCoVs and β-HCoVs, which shall provide useful information for the development of effective preventives to better prepare for outbreaks of ‘CoV-X’ in the future.

## Methods

### Gene cloning, protein expression and purification

The S ectodomain (17–1115 aa), S1 (17–560 aa), S2 (575–1115 aa), NTD (17–265 aa) and RBD (294–435 aa) of 229E (Uniprot ID: P15423) and the S ectodomain (16–1291 aa) of NL63 (Uniprot ID: Q6Q1S2) were each cloned into a modified pFastBac1 vector (Invitrogen), with N-terminal gp67 signal peptide and C-terminal T4 trimerization motif followed by TEV cleavage site and 6 × His tag. Mutants of 229E S protein and NTD were constructed by site-directed mutagenesis and verified by DNA sequencing. Expression and purification of the wild-type proteins and mutants were performed using the method described below.

The expression of 229E S ectodomain was performed using the Bac-to-Bac system (Invitrogen) according to the manufacturer’s protocol. Briefly, baculovirus was prepared by transfecting freshly prepared bacmids into Sf9 cells using FuGENE HD (Promega) and used to infect suspension High Five cells at a MOI of 5 to 10. Medium supernatant of infected High Five cells was harvested after 72 h and supplemented with 1 mM PMSF before being loaded onto an Excel Ni-NTA column (GE Healthcare). Elution fractions containing 229E S trimer were then pooled and further purified by two sequential gel filtration chromatography on a Superpose 6 10/300 GL column (GE Healthcare) equilibrated with 20 mM Tris 8.0, 150 mM NaCl. Gel filtration peak fractions containing the 229E S trimer were then concentrated and stored at −80 °C until further use. The S ectodomain of NL63 was prepared with the same strategy. S1, S2, NTD and RBD of 229E were expressed and purified in similar ways, except that their gel filtration steps were carried out on Superdex 200 10/300 column (GE Healthcare).

The soluble ectodomain (66–967 aa) of hAPN (Uniprot ID: P15144) was cloned into pACEBac1 vector for insect cells expression. An N-terminal honeybee melittin (HBM) signal peptide and a C-terminal 6 × His tag were added to facilitate protein expression and purification. The hAPN protein was purified in similar ways as described above. Purified hAPN was concentrated and stored in buffer containing 20 mM Tris 8.0, 150 mM NaCl, 5% glycerol at −80 °C until further use.

For the expression of Fabs, DNA encoding heavy chain and light chain sequence were amplified by PCR using phagemid DNA as template and subcloned into a modified pRH2.2 expression vector containing a 23-residue signaling peptide (MKKNIAFLLASMFVFSIATNAYA) at the N-terminal and a 6 × His tag at the C-terminal. The Fab constructs were then transformed into *Escherichia coli* BL21 (DE3) for protein expression. Briefly, the cells were cultured in Luria-Bertani (LB) medium at 37 °C until the OD600 reached 0.8. The cultures were then incubated for 16 h at 20 °C in the presence of 0.5 mM isopropylthio-beta-D-galactoside (IPTG) before being harvested by centrifugation at 5000 *g* for 5 min. Cell pellets were then resuspended in PBS buffer, pH 7.4, and lysed using a NANO homogenize machine (ATS Engineering Limited) at 800 bar, 4 °C. After centrifugation, the supernatants were mixed with Talon metal-affinity resins (Clontech) and eluted with a linear gradient of 5–500 mM imidazole. Elution fractions were then further purified on HiTrap Protein A HP column (GE Healthcare) before being loaded onto a Superdex 200 10/300 GL gel filtration column (GE Healthcare). Gel filtration peak fractions containing the target antibodies were pooled, concentrated, and stored at −80 °C for further use.

For antibodies in full-length IgG1 format, heavy chain and light chain sequences were separately cloned into pFuse-hIgG1-Fc2 and pFuse2ss-CLIg-hK expression vectors (InvivoGen). IgGs were expressed through co-transfecting heavy and light chain plasmids into HEK293F cells at 1:1 molar ratio. The transfected cells were cultured for another 6 days before being harvested. Antibodies in the medium were captured with HiTrap Protein A HP column (GE Healthcare) and further purified by size exclusion chromatography. Purified antibodies were concentrated and stored in PBS buffer (pH 7.4) at −80 °C.

### Selection and affinity maturation of antibodies against 229E S trimer

Phage selections were conducted using a validated phage-displayed, synthetic antigen-binding fragment (Fab) library with a diversity of 3 × 10^10^ unique clones, as described with the following modifications^40^. In brief, wells of a 96-well capture plate (ThermoFisher Scientific) were coated overnight with a 5 μg/mL solution of neutravidin (Manufacturer) in PBS at 4 °C, then blocked with 0.2% BSA solution for 1 h before being washed 4 times with PBS, 0.05% Tween (PT) buffer. In parallel, phage library was incubated with biotinylated 50 nM 229E S trimer for 2 h, then transferred to neutravidin-coated capture plates. After 15 min incubation, plates were washed 8 times with PT buffer and phage clones were eluted and amplified as previously reported^40^. The variable regions of clones that bound to immobilized 229E S protein but not to BSA control protein by ELISA^62^, were PCR amplified and subjected to DNA sequencing to decode antibody variable regions.

For affinity maturation of C04, library templates were generated by introducing stop sites into CDRs to be randomized using mutagenic oligonucleotides via Kunkel’s method^63^. CDRs of template DNA were then soft-randomized by oligonucleotide-directed mutagenesis using 70:10:10:10 mixtures of nucleotides biased toward the C04 parental sequence using the same methods. Mutated, covalently closed circular DNA was then electroporated into electrocompetent *E. coli* SS320 cells and phages were amplified as previously reported^64^ to generate maturation libraries. Selections were conducted as described above with diminishing concentrations of biotinylated RBD in each subsequent round of selection.

### Package of 229E Pseudovirus (PsV)

Coronavirus PsV was generated as previously reported^65^. In brief, HEK293T cells were co-transfected with plasmid pcDNA3.1-229E-spike and HIV backbone plasmid pNL4.3-HIV-luc. 48 hours later, cell supernatants containing 229E PsV were collected and stored in −80 °C.

### Assessment of anti-229E PsV activity of antibodies

Anti-229E PsV activity of antibodies was measured as previously reported^66^. Briefly, antibodies were first serially diluted and co-incubated with 229E PsV for 30 min. Next, mixtures containing 229E PsV and diluted antibodies were added into Caco2 cells in 96-well plates. Fresh DMEM containing 2% FBS was added after 12 h. Another 48 h later, luciferase activity was measured and IC_50_ values were calculated.

### Surface plasmon resonance (SPR)

SPR experiments were performed using a Biacore 8 K (GE Healthcare). All assays were performed with a running buffer containing 10 mM HEPES pH 7.4, 150 mM NaCl, 3 mM EDTA and 0.01% v/v Tween-20 at 25 °C. For affinity determinations of most antibodies, recombinant 229E S protein, S1, RBD, NTD and NL63 S protein were each immobilized to a single flow cell on a CM5 sensor chip (GE Healthcare). Three samples containing only running buffer were injected over both sample and reference flow cells, followed by 2-fold serial dilutions of purified antibodies (30 μL/min, association 240 s, dissociation 540 s). To measure the binding affinity of Fab F12 to 229E S2 or NL63 S protein, serial dilutions of recombinant 229E S2 or NL63 S were flowed over immobilized Fab F12 instead. All the binding data were double referenced by blank cycle and reference flow cell subtraction. The resulting sensorgrams were fit to a 1:1 Langmuir binding model using the Biacore Insight Evaluation Software (GE Healthcare).

For competitive binding assays of antibody pairs, S1 protein was immobilized onto the CM5 sensor chip as described above. Then, different antibodies were pairwise tested using the dual injection feature. Briefly, the primary antibody was flowed over the immobilized S1 protein surface for 400 s at 20 μL/min to achieve saturation, followed by a second injection of a mixture containing primary and secondary antibody pairs. The response units were analyzed using the same software as mentioned above.

### Hydrogen–deuterium exchange mass spectrometry (HDX-MS)

Amide hydrogen exchange of 229E S or 229E S-antibody complexes was performed manually as previously reported^67^. Briefly, amide hydrogen exchange of 229E S trimer alone were started by diluting 2 μL protein sample at 30 μM into 18 μL D_2_O buffer (20 mM Tris, pD 8.0, 150 mM NaCl) at 20 °C. At different timepoints (0 s, 10 s, 100 s, 1,000 s and 10,000 s), the labeling reaction was quenched by the addition of chilled quench buffer to a final concentration of 1% FA, pH 3.0, 1.6 M urea, 100 mM TCEP and immediately frozen in liquid nitrogen. For the HDX-MS of S protein in the presence of Fab F12, a 6-fold molar excess of Fab F12 was incubated with 229E S trimer for 60 minutes on ice. 2 μL mixture was then labeled, quenched and flash frozen as above. All frozen samples were stored at −80 °C until analysis. For the HDX-MS of 229E NTD in the absence and presence of Fab D12, 2 μL NTD alone or 2 μL D12-bound NTD was labeled, quenched and flash-frozen as described above. For the rest NTD-directed Abs, the labeling reaction was carried out in similar ways except that only 0 s, 10 s and 10,000 s timepoints were measured to increase experimental throughput. All experiments were independently repeated twice.

The thawed samples were immediately injected into HPLC-MS (Agilent 1100) system equipped with in-line peptic digestion and desalting. The desalted digests were then separated with a Hypersil Gold^TM^ analytical column (ThermoFisher) over an 18 min gradient and directly analyzed with an Orbitrap Fusion mass spectrometer (ThermoFisher). The HPLC system were extensively cleaned with blank injections between samples to minimize any carryover. Peptide identification was performed by tandem MS/MS under orbi/orbi mode. All MS/MS spectra were analyzed using the Proteome Discoverer Software (ThermoFisher). We carried out the initial analysis of the peptide centroids with HD-Examiner v2.3 (Sierra Analytics) and then manually verified every peptide to check retention time, charge state, m/z range and the presence of overlapping peptides. The peptide coverage of 229E S and NTD were found to be 90% and 89% respectively (Supplementary Fig. 7). The relative deuteration levels (%D) of each peptide were automatically calculated by HD-Examiner with the assumption that a fully deuterated sample retains 90% D in current LC setting.

### B-cell epitope prediction

B-cell epitopes of 229E S protein and NL63 S protein were predicted using BepiPred 2.0 algorithm (IEDB, https://www.iedb.org/) with a cutoff of 0.52 (corresponding to a specificity greater than 0.6 and a sensitivity less than 0.4). The predicted epitopes were further curated according to the following criteria: (1) whether they are at least 5 aa long; (2) whether they are solvent-exposed in one-RBD-up 229E or NL63 S trimer; (3) whether they are close (within 5 Å) to known shielding glycans on 229E or NL63 S trimer. Curated prediction results were labeled in corresponding structures using program Pymol (The PyMOL Molecular Graphics System, Version 2.1 Schrödinger, LLC).

### Molecular docking

Models for Fabs F12 and D12 were derived by homology modeling using Swiss Model website^68^. The model of 229E S trimer with one RBD ‘up’ were manually adjusted in Chimera^69^, using SARS-CoV-2 S trimer with one RBD ‘up’ (PDB:6VYB) as reference. F12 and D12 models were docked onto 229E S trimer in all-RBD-down conformation (PDB:6U7H) or 229E S trimer in ‘one RBD up’ conformation respectively using PatchDock^70^. For PatchDock simulations, antibodies and 229E S trimer were set as receptor and ligand respectively under antibody-antigen complex mode, and D12 or F12 epitopes on 229E S as double-defined by HDX-MS and mutagenesis experiments were provided to PatchDock algorithm as ligand binding sites. Docking models were further refined with FireDock^71^.

### Crystallization and X-ray Structure Determination

Purified RBD protein was mixed with Fab C04 or Fab S11 at a molar ratio of 1:1.2 and incubated overnight at 4 °C to form RBD/C04 or RBD/S11 complex. Crystallization screening of Fab C04, RBD/C04 complex and RBD/S11 complex was carried out using the hanging drop vapor diffusion method at 20 °C. Diffraction-quality crystals of C04 were obtained in condition containing 15% w/v polyethylene glycol 3350, 0.01 M magnesium chloride hexahydrate, 0.005 M nickel (II) chloride hexahydrate and 0.1 M HEPES pH 7.0. Crystals of RBD in complex with C04 were obtained in conditions containing 1.6 M ammonium sulfate, 0.1 M Critic Acid pH 5.0 while crystals of RBD/S11 complex were obtained in conditions containing 3.6 M sodium formate, 10% glycerol. Crystals were harvested with 20% (v/v) glycerol as cryoprotectant and flash-cooled in liquid nitrogen for data collection.

X-ray diffraction data were collected at 0.978 Å wavelength on beamline BL19U1 at the Shanghai Synchrotron Radiation Facility (SSRF) and processed using HKL2000 or Xia2-DIAL program^72,73^. Structures were each solved through molecular replacement (MR) with PHASER^74^, using MR templates derived from PDB deposits 6ATK and 6DF2. Iterative model building and refinement were carried out in COOT and PHENIX, respectively^75,76^. The quality of the final models was analyzed with MolProbity^77^. Data collection and refinement statistics are outlined in Table 1. Ramachandran statistics for structures of Fab C04, RBD-C04 complex and RBD-S11 complex indicate that there is no outliers, the scores of ‘allowed’ are 2.3%, 4.9% and 3.9%, respectively, for the three structures and the scores of ‘favored’ are 97.7%, 95.1% and 96.1% respectively for the three structures.

Figures were prepared using program Pymol (The PyMOL Molecular Graphics System, Version 2.1 Schrödinger, LLC). Epitope and paratope residues, as well as buried solvent-accessible surface area were identified with PDBePISA^78^.

### Negative stain electron microscopy (nsEM)

Protein samples to be examined were diluted to an appropriate concentration and dropped onto a freshly glow-discharged carbon-coated grid. After rinsing twice with buffer (20 mM Tris, 150 mM NaCl, pH 8.0), the grid was stained with 2% uranyl formate (pH 7.0) and then loaded onto a 120 kV TEM for examination. To visualize the C04-induced disassembly of 229E S, 229E S protein was incubated at room temperature for 1 h with or without excess C04. The incubated samples were then prepared as described above and visualized using a FEI Talos C-Twin electron microscope operated at 120 kV. 23–31 representative images for each sample were manually collected at 73,000 ×nominal magnification (1.92 Å/pixel) with a defocus range of −2 to −3 μm. Image processing, particles picking and reference-free 2D classification were all performed in RELION v3.1^79^. Projections of 229E S protomer-C04 model were matched to experimental 2D class averages using EMAN2 command e2classsvsproj.py^80^

### Cryo-EM data collection and image processing

229E S protein (2.4 mg/mL) was incubated with 6-fold molar excess of Fab F12 for 60 min before being applied to a glow-discharged holey carbon grid (Quantifoil, R2/1, 300). The grids were blotted using Vitrobot (ThermoFisher, USA) for 3 s with a force level of −1 at 100% humidity and 8 °C. Blotted grids were immediately plunged into liquid ethane cooled by liquid nitrogen. Cryo-EM data were collected using a 300 kV Titan Krios G2 electron microscope (ThermoFisher, USA) equipped with a K3 Summit direct electron detector (Gatan, USA) in super-resolution mode with a pixel size of 0.53 Å. Each movie was exposed for 2.8 s and dose-fractioned into 40 frames with a total dose of ~50 e^−^/A^2^ on the samples. The defocus values used during data collection varied from 0.8 to 2.5 μm. All images were collected using the SerialEM automated data collection software package. Movies were aligned using MotionCor2^81^ after binning super resolution movies by 2 (1.06 Å /pixel) and the defocus values were estimated by contrast transfer function (CTF) estimation using CTFIND4^82^. Particles were automatically picked using Relion 3.1^79^ from manually selected micrographs. Particles were extracted and subjected to multiple rounds of reference-free 2D classification using cryoSPARC v2.5^83^. The well-defined particle images were retained for further analysis. The orientation bias of the particles precluded 3D reconstruction. Projections of 229E S trimer-F12 docking models were matched to experimental 2D class averages of those well-defined particles using EMAN2 command e2classsvsproj.py^80^.

### Sequence conservation calculation

To calculate the sequence conservation at the 229E NTD antigenic supersite across α-HCoVs, 66 229E and 70 NL63 S protein sequences (please see Supplementary Table 3) were downloaded from the Uniprot website (http://www.uniprot.org) and aligned with Clustal Omega^84^. The positional amino acid frequencies at the supersite were then calculated using the Entropy webserver (https://www.hiv.lanl.gov/content/sequence/ENTROPY) and displayed in WebLogo plots^85^. The positional conservation scores at the supersite were calculated with the ConSeq webserver^86^.

### Statistics and reproducibility

The pseudovirus neutralization data from at least two biological repeats were analyzed in Prism 9 software (GraphPad) using a four-parameter logistic regression model. Data are shown as individual data points and mean ± SD.

### Reporting summary

Further information on research design is available in the Nature Research Reporting Summary linked to this article.

## Supplementary information

Supplementary Information

Description of Additional Supplementary Files

Supplementary Data 1

Reporting Summary

## Data Availability

The coordinates of C04, 229E RBD in complex with C04 or S11 have been deposited to the PDB under accession numbers 7VMZ, 7VN9 and 7VNG respectively. Additional data that contributed to this study are present in the Supplementary Information and source data for graphs and charts can be found in Supplementary Data 1 associated with this article. The uncropped and unedited gel images are included in Supplementary Fig. 8. All other data related to this paper may be requested from the corresponding authors.
