# Supplementary material for: Antigenic mapping reveals sites of vulnerability on α-HCoV spike protein
Source: Commun Biol. 2022 Nov 4;5:1179. doi: 10.1038/s42003-022-04160-8 (PMC9636267; doi:10.1038/s42003-022-04160-8)
Supplement: Supplementary file 1 — Supplementary Information [file 42003_2022_4160_MOESM1_ESM.pdf]

Supplementary Information

Antigenic Mapping Reveals Sites of Vulnerability on α-HCoV Spike protein

Jiangchao Xiang[1,2,3,†], Jie Su[1,†], Qiaoshuai Lan[4,†], Wenwen Zhao[1], Yu Zhou[1], Youwei Xu[1], Jun Niu[1], Shuai Xia[4], Qilian Qi[1], Sachdev Sidhu[5], Lu Lu[4,✉], Shane Miersch[5,✉], Bei Yang[1,6,✉]

*Corresponding author. Email: lul@fudan.edu.cn (L.L.), shane.miersch@utoronto.ca (S.M.), yangbei@shanghaitech.edu.cn (B.Y.)

This PDF file includes:

Supplementary Figures 1 to 8
Supplementary Tables 1 to 3
Supplementary References 1 to 7

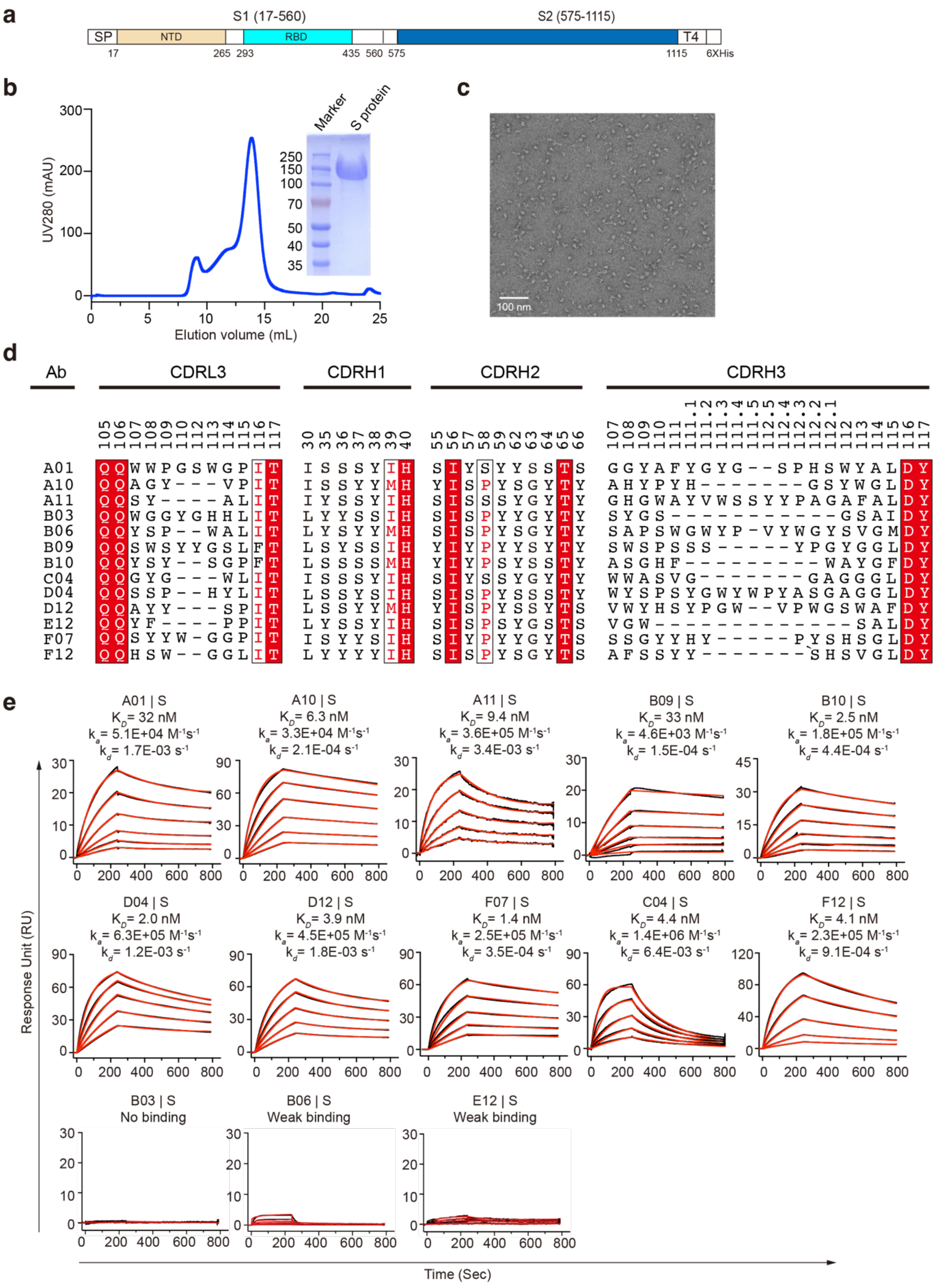

Supplementary Figure 1. Selection and validation of antibodies against 229E S trimer. a Schematic diagram depicting the domain architecture of constructs encoding 229E S protein. The start and end number of each domain are labeled. SP, signal peptide; T4, T4 trimerization

domain. **b, c** Size-exclusion chromatography profile **(b)** and representative nsEM image **(c)** indicate the purity, homogeneity, and trimeric nature of intact 229E S trimer used for antibody selection. Inset: Colloidal Coomassie-staining of size-exclusion chromatography peak fractions. **d** Alignment of the CDR sequences for putative 229E S trimer-binding antibodies. CDRs are numbered according to IMGT numbering [1]. **e** The binding of 13 identified Fabs to 229E S trimer were validated by SPR. Two-fold serial dilutions were injected onto immobilized 229E S trimer. For each Fab, SPR experiments were performed at least twice and kinetic data from one representative experiment was shown. Raw SPR profiles were shown as black traces and global kinetic fits were presented as red lines.

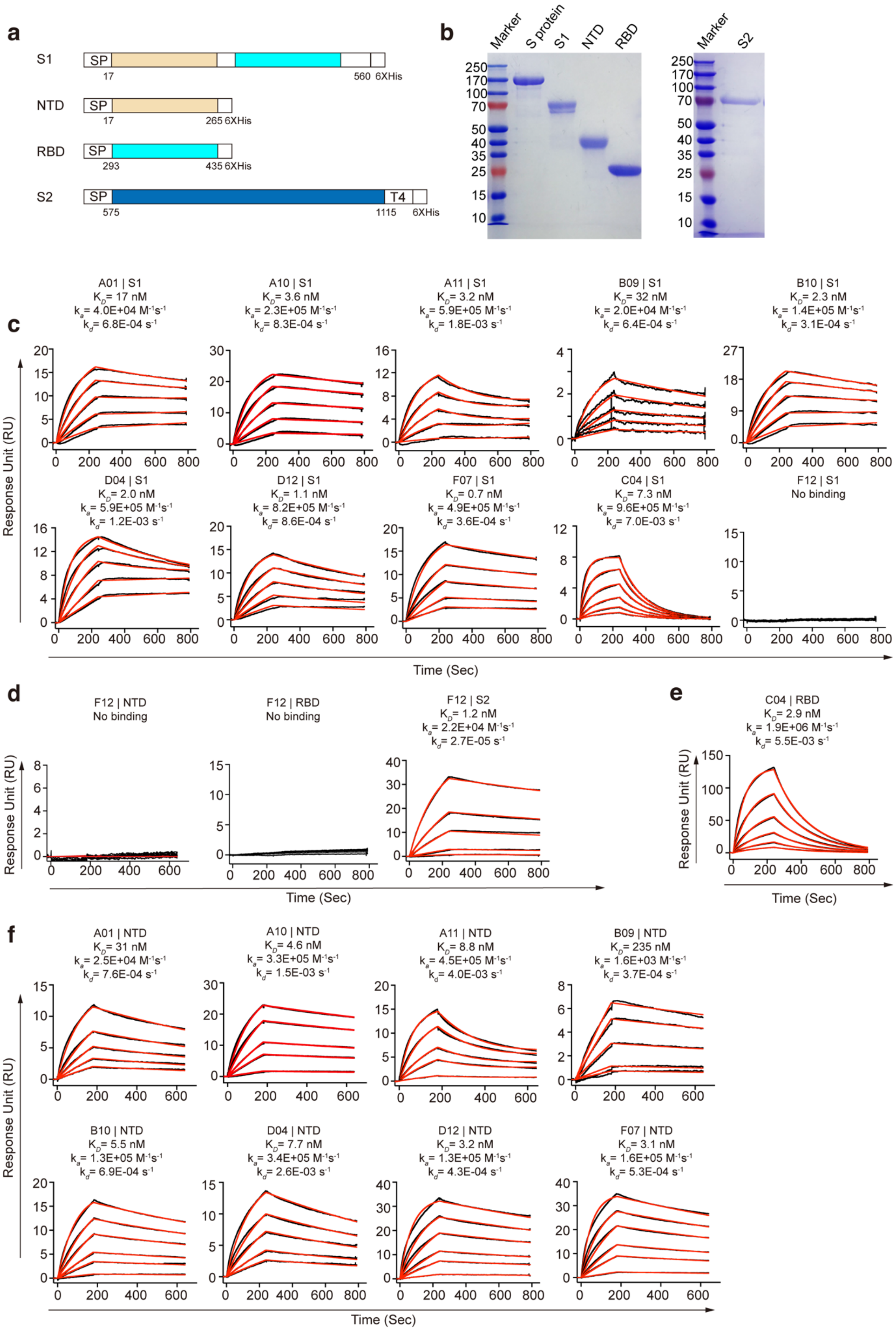
a
S1
SP
17
560 6XHis
NTD
265 6XHis
RBD
293
435 6XHis
S2
T4
575
1115
b
Marker
S protein
NTD
c
A01 | S1
A10 | S1
A11 | S1
B09 | S1
B10 | S1
D04 | S1
D12 | S1
F07 | S1
C04 | S1
F12 | S1
No binding
Response Unit (RU)
Time (Sec)
d
F12 | NTD
No binding
F12 | RBD
No binding
F12 | S2
e
C04 | RBD
f
A01 | NTD
A10 | NTD
A11 | NTD
B09 | NTD
B10 | NTD
D04 | NTD
D12 | NTD
F07 | NTD

Supplementary Figure 2. Antibody classification via SPR. a Schematic diagram depicting the domain architecture of constructs encoding 229E S1 subunit, NTD, RBD and S2 subunit. The start and end numbers of each domain are labeled. SP, signal peptide; T4, T4 trimerization domain. **b** Colloidal Coomassie-staining SDS-PAGE gel of 229E S, S1, NTD, RBD and S2 used for SPR experiments. **c** SPR profiles of indicated Fabs to immobilized 229E S1. **d** SPR profiles of Fab F12 to NTD, RBD and S2. **e** SPR profiles of Fab C04 to RBD. **f** SPR profiles of indicated Fabs to NTD. **(c-f)** For each Fab, SPR experiments were performed at least twice and kinetic data from one representative experiment was shown. Raw SPR profiles were shown as black traces and global kinetic fits were presented as red lines.

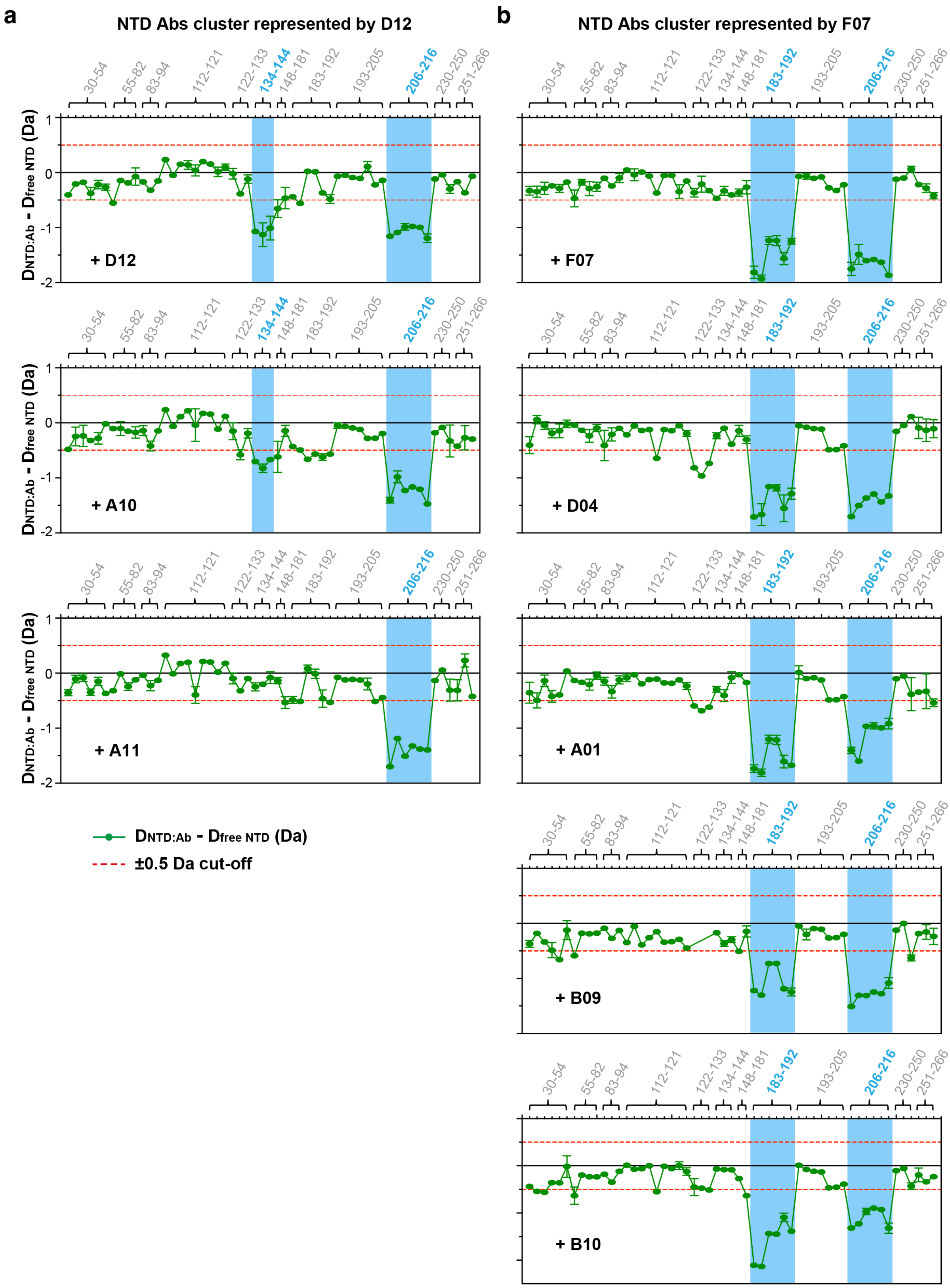

Supplementary Figure 3. Mapping the epitopes of all NTD antibodies with HDX-MS. a, b Deuteron incorporation difference plots (NTD:Fab - free NTD) for 229E NTD peptides (t=10 s). Each point denotes an identified peptide of 229E NTD, displayed from the N to the

C terminus. A positive difference indicates Fab-binding induced increase in solvent accessibility, and a negative difference indicates Fab-binding induced protection form HDX. Peptides that manifested significant HDX protection were indicated with light blue regions and labeled in bold. Some of the antibodies manifested HDX protection profiles similar to that of D12 **(a)**, while others exhibited HDX protection profiles similar to that of F07 **(b)**. Dashed red lines depict significance thresholds for deuteron incorporation differences (±0.5 Da) [2], points lying within the lines are not significantly different from zero. Standard errors of deuteron incorporation differences between repeated measurements (n=2 biologically independent experiments) are shown as error bars for each peptide.

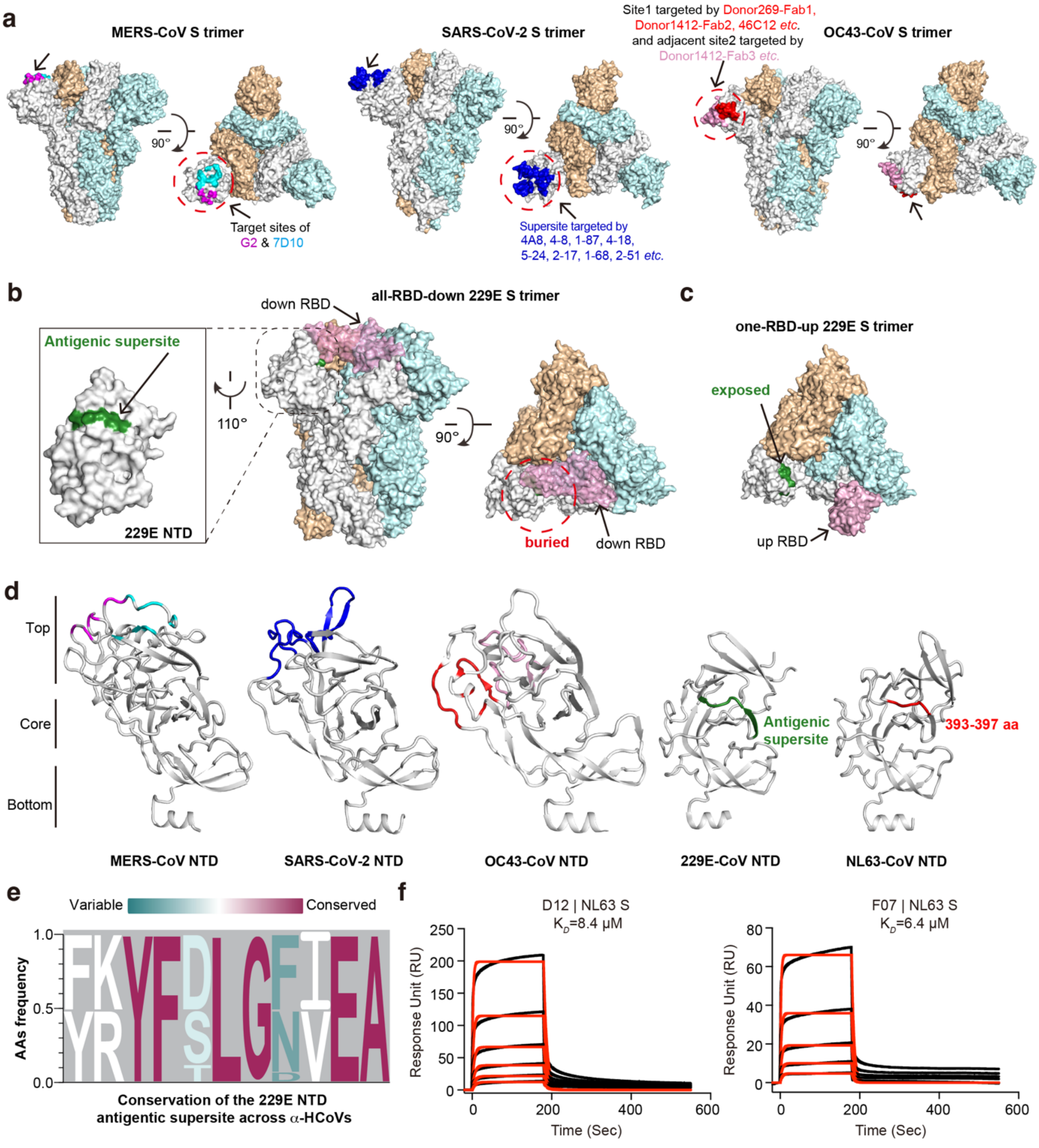

Supplementary Figure 4. The NTD epitopes in α-HCoVs and β-HCoVs. a Left panel: epitopes recognized by antibody G2 and 7D10 [3,4] are highlighted in magenta and cyan respectively on the surface representation of MERS-CoV S trimer (PDB: 6Q04); middle panel: the antigenic supersite recognized by antibodies 4A8, 4-8, 1-87, 4-18, 5-24, 2-17, 1-68 and 2-51 *etc.* [5] are highlighted in blue on the surface representation of SARS-CoV-2 S trimer (PDB:7L2D); right panel: antigenic supersite 1 recognized by Donor269-Fab1, Donor1412-Fab2, 46C12 *etc.* and supersite 2 recognized by Donor1412-Fab3 *etc.* are highlighted in red

and pink on the surface representation of OC43 S trimer (PDB:6OHW) [6,7]. **b, c** An antigenic supersite is also present on the NTD of 229E, which appears partially buried in all-RBD-down 229E S trimer (PDB: 6U7H) **(b)** yet becomes fully exposed in one-RBD-up 229E S trimer **(c)**. The consensus epitope (206-215 aa) of all 229E NTD-directed antibodies was highlighted in dark green on the surface representation of 229E S trimer and NTD (close-up view). **d** The NTDs from β-HCoVs (MERS-CoV, SARS-CoV-2 and OC43) and α-HCoVs (229E and NL63) are depicted as ribbons. The NTD epitopes of MERS-CoV, SARS-CoV-2 and OC43 were colored as in **(a)**, the antigenic supersite in 229E NTD was colored dark green as in **(b)**. A strong B-Cell epitope (393-397 aa, predicted with BepiPred 2.0) in the NTD of NL63 was colored in red to highlight its positional similarity to the antigenic supersite in 229E NTD. The NTDs from β-HCoVs could be separated into top, core, and bottom layers. While the bottom layer appears structurally conserved between α-and β-HCoVs, the top layer is quite diverse across genera. **e** The positional amino acid frequencies and conservation scores at the proposed NTD antigenic supersite were calculated across 66 229E and 70 NL63 S sequences and presented as WebLogo plot. The S protein accession IDs and sequences are stored in Supplementary Table 4. Letter height represents AA frequencies. Letters were colored according to the given scale. **f** Representative SPR sensorgrams of Fabs D12 and F07 to immobilized NL63 S protein. Raw SPR profiles were shown as black traces and global kinetic fit were presented as red lines. All SPR experiments were independently performed at least three times and representative profiles from one experiment were shown. The K_D values derived from equilibrium analysis (D12: 3.8 ± 0.2 μM and F07: 3.9 ± 0.3 μM) shown in Fig.2d are similar to those obtained from kinetic analysis (D12: 8.4 ± 0.4 μM and F07: 6.4 ± 0.3 μM), both indicating weak yet definite binding between NL63 S protein and the two Fabs.

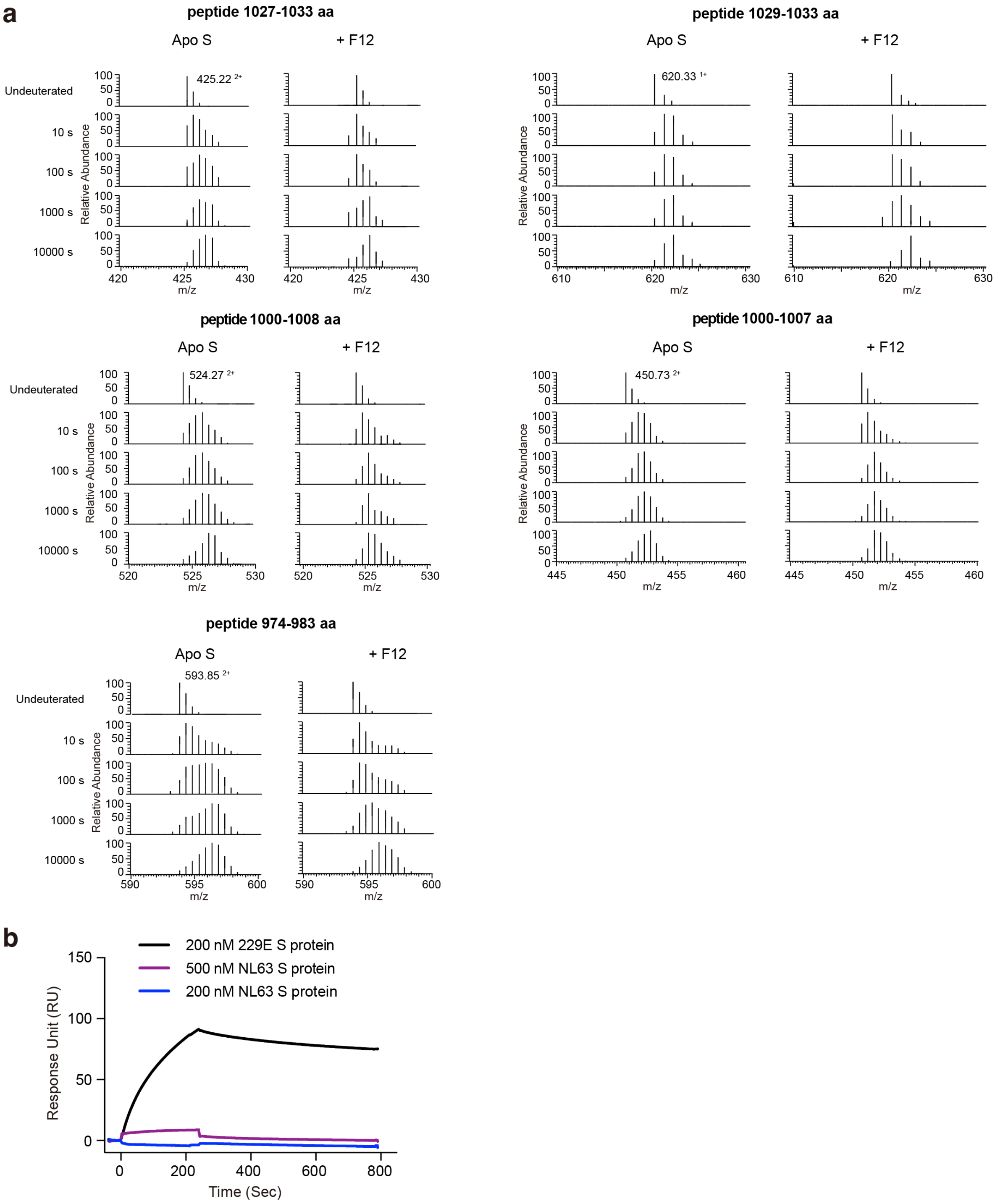

Supplementary Figure 5. Raw spectras of 229E S2 peptides that manifested significant HDX protection in the presence of Fab F12. a Mass spectras of indicated peptides at different labeling timepoints, with the mass spectras of undeuterated samples shown as controls. **b** F12 lacks cross-reactivity to NL63 S protein. SPR profiles indicates no binding between F12 and NL63 S protein. 229E S protein served as positive control. All SPR

experiments were independently performed at least twice and representative profiles from one experiment were shown.

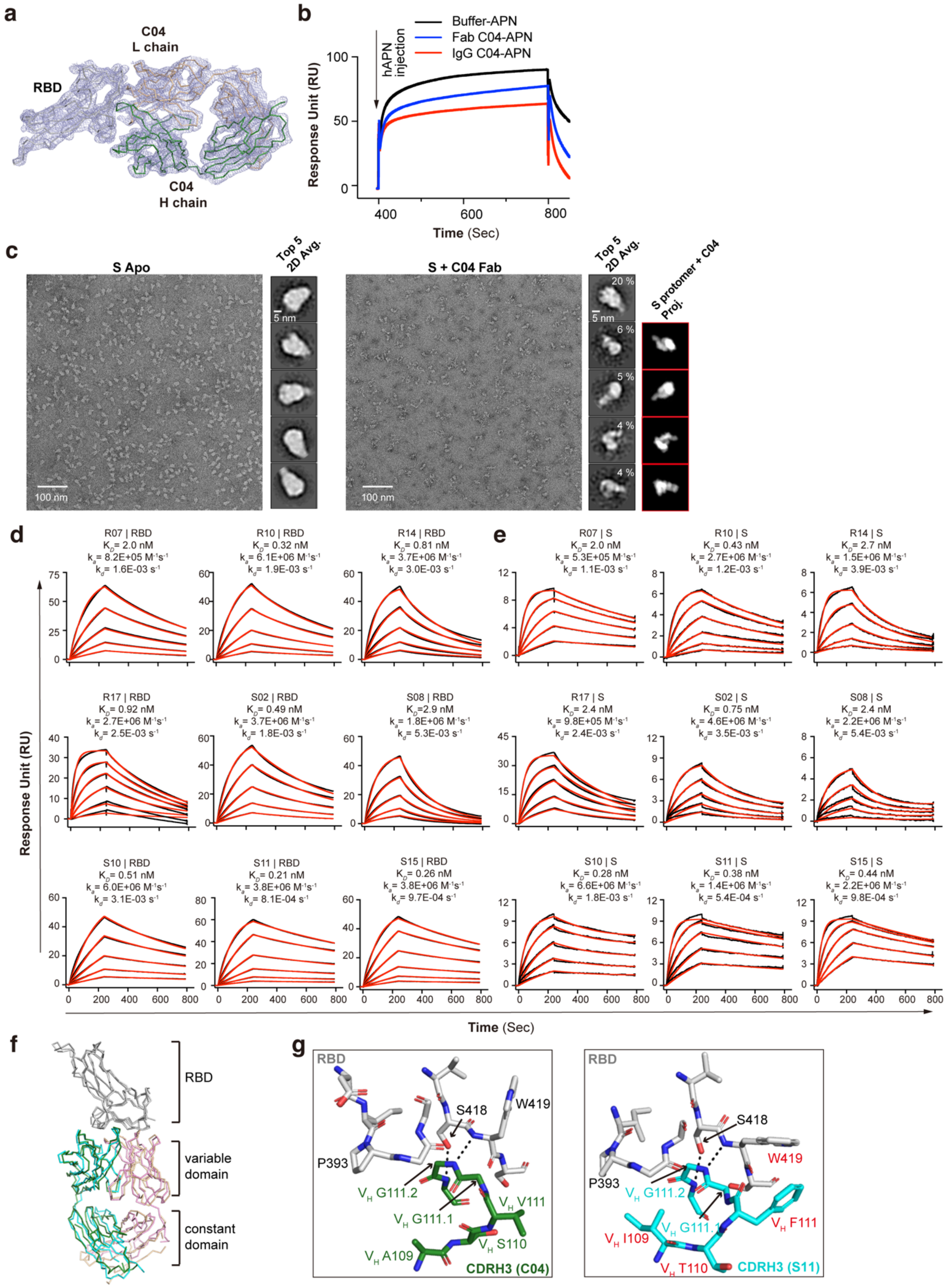

Supplementary Figure 6. C04 interferes with the binding of hAPN to 229E S trimer and induces S trimer disassembly. **a** 2Fo-Fc electron density map illustrating the overall quality of the RBD-C04 complex structure. The density map is colored purple and contoured at 1.5

σ. **b** Competitive SPR results indicate that pre-binding of C04 in IgG or Fab format would slightly interfere with the binding of hAPN. Immobilized 229E S trimer was saturated with C04 in either Fab or IgG format before the injection of recombinant hAPN proteins to evaluate antibody-mediated blockade of S protein: receptor interactions. **c** Representative nsEM micrographs of 229E S protein in the absence (left) and presence (right) of C04 illustrating the disassembly of 229E S trimers induced by C04 binding over 1 hr. Shown to the right of each nsEM micrograph are the top 5 reference-free 2D class averages of corresponding samples. To examine the correspondence between nsEM 2D class averages and the model of C04 bound-229E protomer, representative 2D projections of this model were also shown. In the presence of C04, at least 19% (6%+5%+4%+4%) of the 229E S trimer have disassembled into C04 bound-229E protomers while ~20% of the particles remained as intact free S trimers. **d, e** Binding curves of affinity-matured antibodies to immobilized 229E RBD **(d)** and 229E S trimer **(e)**. Data are shown as black lines and the best fits of the data to a 1:1 binding model are shown in red. **f** Structural superimposition of the RBD-C04 and the RBD-S11 complexes, 229E RBD was depicted as grey ribbons, the heavy and light chains of C04 were colored dark green and wheat and that of S11 were colored cyan and pink. **g** Close-up views illustrating the interactions between 229E RBD and CDRH3 of C04 (left panel) or S11 (right panel). Interacting residues were shown as ball and stick models and labeled. H-bonds are represented with dashed black lines. Note that interface residues that manifested obvious positional shifts after affinity maturation were highlighted with red labels. **(b, d, e)** All SPR experiments were independently performed at least twice and representative profiles from one experiment were shown.

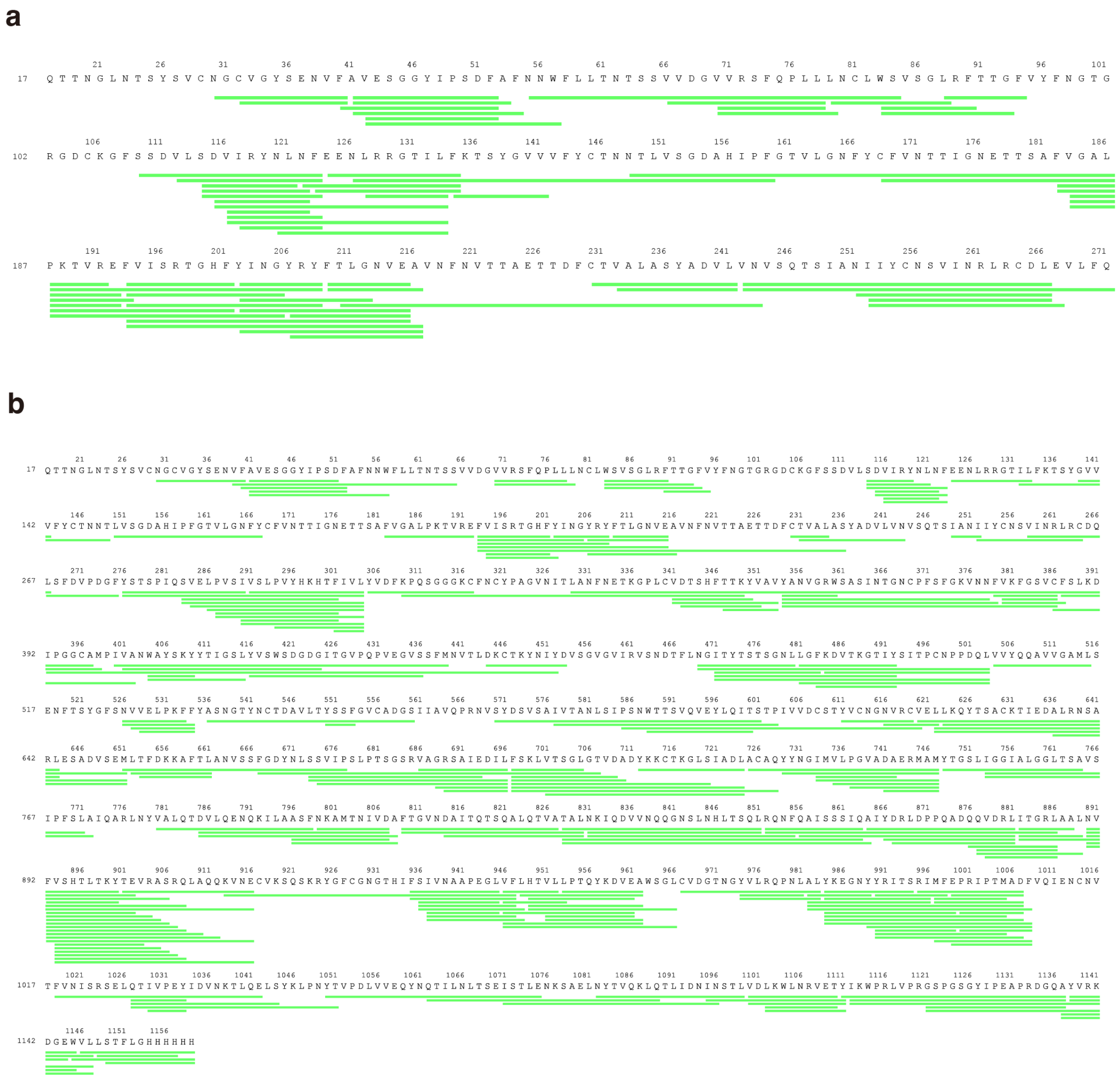

Supplementary Figure 7. HDX-MS peptides coverage of 229E NTD and 229E S. The HDX-MS peptide coverage map for 229E NTD **(a)** and 229E S **(b)**. Green lines below the protein sequence represent the digested peptides that were identified and analyzed in this study.

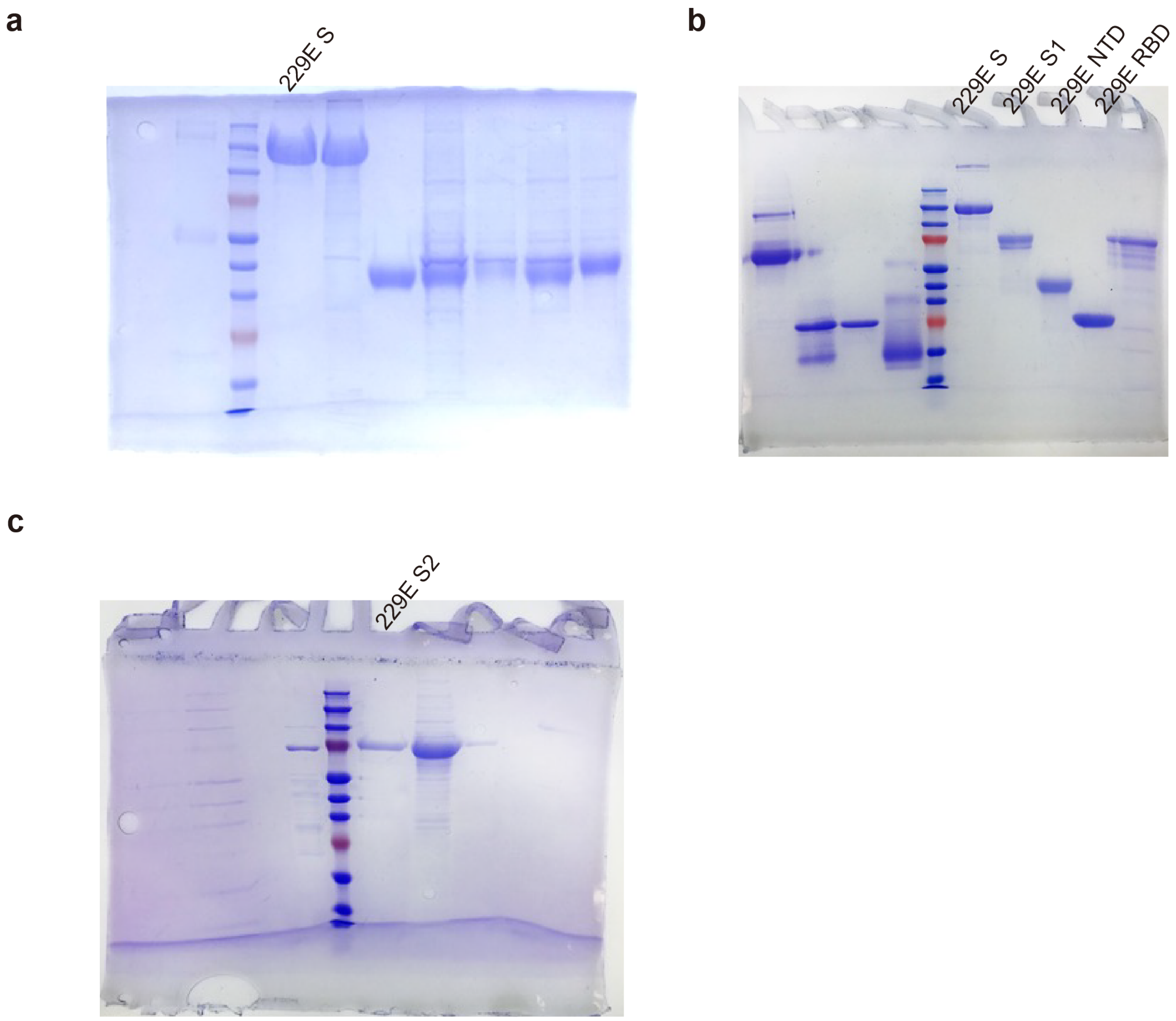

Supplementary Figure 8. Uncropped gel images used in this study. a The uncropped and unedited gel image for Supplementary Figure 1b. **b, c** The uncropped and unedited gel images for Supplementary Figure 2b.

Supplementary Table 1. Interactions between indicated antibodies and 229E NTD or its indicated mutants.

Analytes	F07		D12	
	K_D (nM)	Relative affinity	K_D (nM)	Relative affinity
Wild-type 229E NTD	15.4	100%	23.0	100%
Y208A	48.6	31.7%	58.1	39.6%
F209A	89.0	16.9%	88.7	25.9%
T210A	368	4.18%	52.0	44.2%
L211A	90.2	17.1%	87.5	26.3%
N213A	14.5	100%	39.6	58.1%
V214A	22.0	70.0%	34.6	66.5%
210A/211A/ 213A/214A	N.B.[1]	0	N.B.[1]	0

[1]N.B. indicates no binding.

Supplementary Table 2. Interactions between Fab F12 and 229E S protein or its indicated mutants.

**Analytes**		**K_D (nM)**	**Relative affinity**
Wild-type 229E S protein		10.1	100%
1000-1007 aa	I1002A	27.0	37.4%
	T1004A	13.5	74.8%
	M1005A	N.B.[1]	0
	I1002A/T1004A/M1005A	N.B.[1]	0
1027-1033 aa	Q1027A	11.7	86.3%
	V1030A	N.B.[1]	0
	P1031A	18.8	53.7%
	E1032A	N.B.[1]	0
	Y1033A	19.9	50.8%
	V1030A/ P1031A/ E1032A/ Y1033A	N.B.[1]	0

[1]N.B. indicates no binding.

Supplementary Table 3. Uniprot accessions and amino acid sequences at the proposed NTD antigenic supersite for 66 229E and 70 NL63 S protein.

**Uniprot accession ID**	**Sequences at the proposed supersite**
tr|A0A7M3V771|A0A7M3V771_CVH22	**YRYFSLGDVEA**
tr|A0A891F0R9|A0A891F0R9_CVH22	**YRYFSLGNVEA**
tr|A0A5B9MYJ0|A0A5B9MYJ0_CVH22	**YRYFSLGNVEA**
tr|A0A5C2D4L1|A0A5C2D4L1_CVH22	**YRYFSLGNVEA**
tr|A0A891F0C9|A0A891F0C9_CVH22	**YRYFSLGNVEA**
tr|A0A6M6LUN0|A0A6M6LUN0_CVH22	**YRYFSLGDVEA**
tr|A0A6M6M615|A0A6M6M615_CVH22	**YRYFSLGNVEA**
tr|A0A223FUI6|A0A223FUI6_CVH22	**YRYFSLGDVEA**
tr|A0A891EZU3|A0A891EZU3_CVH22	**YRYFSLGDVEA**
tr|A0A6M6M126|A0A6M6M126_CVH22	**YRYFSLGDVEA**
tr|A0A384R9G0|A0A384R9G0_CVH22	**YRYFSLGNVEA**
tr|A0A384RNP7|A0A384RNP7_CVH22	**YRYFSLGNVEA**
tr|A0A384RBT4|A0A384RBT4_CVH22	**YRYFSLGNVEA**
tr|A0A384RK26|A0A384RK26_CVH22	**YRYFSLGNVEA**
tr|A0A2S1PV28|A0A2S1PV28_CVH22	**YRYFSLGNVEA**
tr|A0A1L7B908|A0A1L7B908_CVH22	**YRYFSLGDVEA**
tr|A0A1W6DZS3|A0A1W6DZS3_CVH22	**YRYFSLGDVEA**
tr|A0A1Y0EV30|A0A1Y0EV30_CVH22	**YRYFSLGDVEA**
tr|A0A059SGA9|A0A059SGA9_CVH22	**YRYFTLGNVEA**
tr|H1AG29|H1AG29_CVH22	**YRYFTLGNVEA**
sp|P15423|SPIKE_CVH22	**YRYFTLGNVEA**
tr|Q1HVM6|Q1HVM6_CVH22	**YRYFTLGNVEA**
tr|Q1HVL9|Q1HVL9_CVH22	**YRYFTLGNVEA**
tr|Q1HVM0|Q1HVM0_CVH22	**YRYFTLGNVEA**
tr|Q1HVM3|Q1HVM3_CVH22	**YRYFTLGNVEA**
tr|Q1HVM5|Q1HVM5_CVH22	**YRYFTLGNVEA**
tr|A0A384QXL8|A0A384QXL8_CVH22	**YRYFSLGNVEA**
tr|A0A384RHP9|A0A384RHP9_CVH22	**YRYFSLGNVEA**
tr|A0A384RBT3|A0A384RBT3_CVH22	**YRYFSLGNVEA**
tr|J9UWK8|J9UWK8_CVH22	**YRYFSLGNVEA**
tr|A0A384RJY2|A0A384RJY2_CVH22	**YRYFSLGNVEA**
tr|A0A384RJX8|A0A384RJX8_CVH22	**YRYFSLGNVEA**
tr|A0A1Z1R0Z6|A0A1Z1R0Z6_CVH22	**YRYFSLGDVEA**
tr|A0A384QXM1|A0A384QXM1_CVH22	**YRYFSLGNVEA**
tr|A0A384R9G2|A0A384R9G2_CVH22	**YRYFSLGNVEA**
tr|A0A891F095|A0A891F095_CVH22	**YRYFSLGNVEA**
tr|A0A384R2L9|A0A384R2L9_CVH22	**YRYFSLGNVEA**
tr|A0A384RJY8|A0A384RJY8_CVH22	**YRYFSLGNVEA**

tr|A0A384RBT6|A0A384RBT6_CVH22	**YRYFSLGNVEA**
tr|H1AG33|H1AG33_CVH22	**YRYFSLGNVEA**
tr|A0A384RNQ9|A0A384RNQ9_CVH22	**YRYFSLGNVEA**
tr|A0A384QXM2|A0A384QXM2_CVH22	**YRYFSLGNVEA**
tr|A0A384QXY4|A0A384QXY4_CVH22	**YRYFSLGNVEA**
tr|H1AG32|H1AG32_CVH22	**YRYFSLGNVEA**
tr|H1AG31|H1AG31_CVH22	**YRYFSLGNVEA**
tr|H1AG30|H1AG30_CVH22	**YRYFSLGNVEA**
tr|Q1HVL0|Q1HVL0_CVH22	**YRYFSLGNVEA**
tr|Q1HVL1|Q1HVL1_CVH22	**YRYFSLGNVEA**
tr|Q1HVK5|Q1HVK5_CVH22	**YRYFSLGNVEA**
tr|Q1HVK4|Q1HVK4_CVH22	**YRYFSLGNVEA**
tr|Q1HVK3|Q1HVK3_CVH22	**YRYFSLGNVEA**
tr|A0A384RK44|A0A384RK44_CVH22	**YRYFSLGNVEA**
tr|Q1HVK6|Q1HVK6_CVH22	**YRYFSLGNVEA**
tr|Q1HVK9|Q1HVK9_CVH22	**YRYFSLGNVEA**
tr|A0A7M4C333|A0A7M4C333_CVH22	**YRYFSLGNVEA**
tr|J9VBQ8|J9VBQ8_CVH22	**YRYFSLGNVEA**
tr|Q1HVL7|Q1HVL7_CVH22	**YRYFTLGNVEA**
tr|Q1HVM1|Q1HVM1_CVH22	**YRYFTLGNVEA**
tr|Q1HVL8|Q1HVL8_CVH22	**YRYFTLGNVEA**
tr|S5YNN4|S5YNN4_CVH22	**YRYFSLGNVEA**
tr|S5YNL7|S5YNL7_CVH22	**YRYFTLGNVEA**
tr|S5YGT1|S5YGT1_CVH22	**YRYFTLGNVEA**
tr|S5YAI3|S5YAI3_CVH22	**YRYFSLGNVEA**
tr|Q1HVL3|Q1HVL3_CVH22	**YRYFTLGNVEA**
tr|Q1HVL4|Q1HVL4_CVH22	**YRYFTLGNVEA**
tr|S5YGU2|S5YGU2_CVH22	**YRYFTLGNVEA**
tr|A0A2P1E905|A0A2P1E905_CVHNL	**FKYFDLGFIEA**
tr|A0A513ZT30|A0A513ZT30_CVHNL	**FKYFDLGFIEA**
tr|A0A2L0WQE1|A0A2L0WQE1_CVHNL	**FKYFDLGFIEA**
tr|Q06Y16|Q06Y16_CVHNL	**FKYFDLGFIEA**
tr|A0A5K7VWR5|A0A5K7VWR5_CVHNL	**FKYFDLGFIEA**
tr|A0A1Y0EV12|A0A1Y0EV12_CVHNL	**FKYFDLGFIEA**
tr|A0A5B9BGI8|A0A5B9BGI8_CVHNL	**FKYFDLGFIEA**
tr|A0A5B9BHU7|A0A5B9BHU7_CVHNL	**FKYFDLGFIEA**
tr|A0A5K7W0I8|A0A5K7W0I8_CVHNL	**FKYFDLGFIEA**
tr|A0A2P1E919|A0A2P1E919_CVHNL	**FKYFDLGFIEA**
tr|U3M7H2|U3M7H2_CVHNL	**FKYFDLGFIEA**
tr|H9EJ16|H9EJ16_CVHNL	**FKYFDLGFIEA**
tr|Q06Y10|Q06Y10_CVHNL	**FKYFDLGFIEA**
tr|A0A2P1E918|A0A2P1E918_CVHNL	**FKYFDLGFIEA**
tr|A0A2P1E926|A0A2P1E926_CVHNL	**FKYFDLGFIEA**

tr|A0A2P1E939|A0A2P1E939_CVHNL	**FKYFDLGFIEA**
tr|A0A2P1E917|A0A2P1E917_CVHNL	**FKYFDLGFIEA**
tr|A0A2P1E937|A0A2P1E937_CVHNL	**FKYFDLGFIEA**
tr|A0A2P1E927|A0A2P1E927_CVHNL	**FKYFDLGFIEA**
tr|A0A7U1BH40|A0A7U1BH40_CVHNL	**FKYFDLGFIEA**
tr|A0A2U8JDM5|A0A2U8JDM5_CVHNL	**FKYFDLGFIEA**
tr|A0A5B9MSJ9|A0A5B9MSJ9_CVHNL	**FKYFDLGFIEA**
tr|U3M7G5|U3M7G5_CVHNL	**FKYFDLGFIEA**
tr|U3M6U5|U3M6U5_CVHNL	**FKYFDLGFIEA**
tr|U3M6S0|U3M6S0_CVHNL	**FKYFDLGFIEA**
tr|H9EJ86|H9EJ86_CVHNL	**FKYFDLGFIEA**
tr|H9EJA0|H9EJA0_CVHNL	**FKYFDLGFIEA**
tr|H9EJ72|H9EJ72_CVHNL	**FKYFDLGFIEA**
tr|H9EJ51|H9EJ51_CVHNL	**FKYFDLGFIEA**
tr|A0A2P1E931|A0A2P1E931_CVHNL	**FKYFDLGFIEA**
sp|Q6Q1S2|SPIKE_CVHNL	**FKYFDLGFIEA**
tr|K4P2Y7|K4P2Y7_CVHNL	**FKYFDLGFIEA**
tr|A0A7U1BH66|A0A7U1BH66_CVHNL	**FKYFDLGFIEA**
tr|A0A2P1E920|A0A2P1E920_CVHNL	**FKYFDLGFIEA**
tr|A0A5B9MUE4|A0A5B9MUE4_CVHNL	**FKYFDLGFIEA**
tr|A0A5B9BIJ4|A0A5B9BIJ4_CVHNL	**FKYFDLGFIEA**
tr|A0A5B9BH95|A0A5B9BH95_CVHNL	**FKYFDLGFIEA**
tr|A0A2P1E915|A0A2P1E915_CVHNL	**FKYFDLGFIEA**
tr|A0A2P1E921|A0A2P1E921_CVHNL	**FKYFDLGFIEA**
tr|A0A2P1E904|A0A2P1E904_CVHNL	**FKYFDLGFIEA**
tr|A0A2P1E912|A0A2P1E912_CVHNL	**FKYFDLGFIEA**
tr|A0A5B9BH24|A0A5B9BH24_CVHNL	**FKYFDLGFIEA**
tr|A0A384R2Q9|A0A384R2Q9_CVHNL	**FKYFDLGFIEA**
tr|A0A384RK30|A0A384RK30_CVHNL	**FKYFDLGFIEA**
tr|A0A1V0E0W0|A0A1V0E0W0_CVHNL	**FKYFDLGFIEA**
tr|A0A1V0PKW6|A0A1V0PKW6_CVHNL	**FKYFDLGFIEA**
tr|A0A1V0PKX8|A0A1V0PKX8_CVHNL	**FKYFDLGFIEA**
tr|A0A1V0E0U5|A0A1V0E0U5_CVHNL	**FKYFDLGFIEA**
tr|H9EJ44|H9EJ44_CVHNL	**FKYFDLGFIEA**
tr|H9EJW4|H9EJW4_CVHNL	**FKYFDLGFIEA**
tr|A0A0P0G321|A0A0P0G321_CVHNL	**FKYFDLGFIEA**
tr|H9EJV2|H9EJV2_CVHNL	**FKYFDLGFIEA**
tr|A0A1L2YVI8|A0A1L2YVI8_CVHNL	**FKYFDLGFIEA**
tr|A0A2P1E930|A0A2P1E930_CVHNL	**FKYFDLGFIEA**
tr|A0A384RHS8|A0A384RHS8_CVHNL	**FKYFDLGFIEA**
tr|A0A384RBV6|A0A384RBV6_CVHNL	**FKYFDLGFIEA**
tr|A0A384QZE9|A0A384QZE9_CVHNL	**FKYFDLGFIEA**
tr|A0A384RHT2|A0A384RHT2_CVHNL	**FKYFDLGFIEA**

tr|I7CMD1|I7CMD1_CVHNL	**FKYFDLGFIEA**
tr|H9EJ37|H9EJ37_CVHNL	**FKYFDLGFIEA**
tr|A0A384QXZ7|A0A384QXZ7_CVHNL	**FKYFDLGFIEA**
tr|A0A2P1E910|A0A2P1E910_CVHNL	**FKYFDLGFIEA**
tr|A0A2P1E932|A0A2P1E932_CVHNL	**FKYFDLGFIEA**
tr|A0A2P1E913|A0A2P1E913_CVHNL	**FKYFDLGFIEA**
tr|A0A0K1D6F6|A0A0K1D6F6_CVHNL	**FKYFDLGFIEA**
tr|A0A2P1E952|A0A2P1E952_CVHNL	**FKYFDLGFIEA**
tr|U3M6Q4|U3M6Q4_CVHNL	**FKYFDLGFIEA**
tr|H9EJ93|H9EJ93_CVHNL	**FKYFDLGFIEA**
tr|A0A1V0PKX1|A0A1V0PKX1_CVHNL	**FKYFDLGFIEA**
tr|A0A2P1E936|A0A2P1E936_CVHNL	**FKYFDLGFIEA**

Supplementary References

1 Lefranc, M.-P. *et al.* IMGT unique numbering for immunoglobulin and T cell receptor variable domains and Ig superfamily V-like domains. *Developmental & Comparative Immunology* **27**, 55-77, doi:https://doi.org/10.1016/S0145-305X(02)00039-3 (2003).

2 Houde, D., Berkowitz, S. A. & Engen, J. R. The utility of hydrogen/deuterium exchange mass spectrometry in biopharmaceutical comparability studies. *J Pharm Sci* **100**, 2071-2086, doi:10.1002/jps.22432 (2011).

3 Zhou, H. *et al.* Structural definition of a neutralization epitope on the N-terminal domain of MERS-CoV spike glycoprotein. *Nat Commun* **10**, 3068, doi:10.1038/s41467-019-10897-4 (2019).

4 Wang, N. *et al.* Structural Definition of a Neutralization-Sensitive Epitope on the MERS-CoV S1-NTD. *Cell Rep* **28**, 3395-3405 e3396, doi:10.1016/j.celrep.2019.08.052 (2019).

5 Cerutti, G. *et al.* Potent SARS-CoV-2 neutralizing antibodies directed against spike N-terminal domain target a single supersite. *Cell Host Microbe* **29**, 819-833 e817, doi:10.1016/j.chom.2021.03.005 (2021).

6 Bangaru, S. *et al.* Structural mapping of antibody landscapes to human betacoronavirus spike proteins. *Sci Adv* **8**, eabn2911, doi:10.1126/sciadv.abn2911 (2022).

7 Wang, C. *et al.* Antigenic structure of the human coronavirus OC43 spike reveals exposed and occluded neutralizing epitopes. *Nat Commun* **13**, 2921, doi:10.1038/s41467-022-30658-0 (2022).
