# Supplementary material for: Antigenic mapping reveals sites of vulnerability on α-HCoV spike protein
Source: Commun Biol. 2022 Nov 4;5:1179. doi: 10.1038/s42003-022-04160-8 (PMC9636267; doi:10.1038/s42003-022-04160-8)
Supplement: Supplementary file 2 — Description of Additional Supplementary Files [file 42003_2022_4160_MOESM2_ESM.pdf]

Description of Additional Supplementary Files

File name: Supplementary Data 1
Description: The source data for Fig. 1c-1e, 2a, 2b, 2d, 2f, 3a, 4g, S1e, S2c-S2f, S4f, S5b, S6b, S6d, and S6e.
