# Supplementary material for: Antigenic mapping reveals sites of vulnerability on α-HCoV spike protein
Source: Commun Biol. 2022 Nov 4;5:1179. doi: 10.1038/s42003-022-04160-8 (PMC9636267; doi:10.1038/s42003-022-04160-8)
Supplement: Supplementary file 4 — Reporting Summary [file 42003_2022_4160_MOESM4_ESM.pdf]

nature portfolio

Corresponding author(s): Bei Yang, Shane Miersch, Lu Lu

Last updated by author(s): Oct 14, 2022

# Reporting Summary

Nature Portfolio wishes to improve the reproducibility of the work that we publish. This form provides structure for consistency and transparency in reporting. For further information on Nature Portfolio policies, see our Editorial Policies and the Editorial Policy Checklist.

## Statistics

For all statistical analyses, confirm that the following items are present in the figure legend, table legend, main text, or Methods section.

| n/a | Confirmed | |
|---|---|---|
| ☐ | ☒ | The exact sample size ($n$) for each experimental group/condition, given as a discrete number and unit of measurement |
| ☐ | ☒ | A statement on whether measurements were taken from distinct samples or whether the same sample was measured repeatedly |
| ☒ | ☐ | The statistical test(s) used AND whether they are one- or two-sided<br>*Only common tests should be described solely by name; describe more complex techniques in the Methods section.* |
| ☒ | ☐ | A description of all covariates tested |
| ☒ | ☐ | A description of any assumptions or corrections, such as tests of normality and adjustment for multiple comparisons |
| ☐ | ☒ | A full description of the statistical parameters including central tendency (e.g. means) or other basic estimates (e.g. regression coefficient) AND variation (e.g. standard deviation) or associated estimates of uncertainty (e.g. confidence intervals) |
| ☒ | ☐ | For null hypothesis testing, the test statistic (e.g. $F$, $t$, $r$) with confidence intervals, effect sizes, degrees of freedom and $P$ value noted<br>*Give P values as exact values whenever suitable.* |
| ☒ | ☐ | For Bayesian analysis, information on the choice of priors and Markov chain Monte Carlo settings |
| ☒ | ☐ | For hierarchical and complex designs, identification of the appropriate level for tests and full reporting of outcomes |
| ☒ | ☐ | Estimates of effect sizes (e.g. Cohen's $d$, Pearson's $r$), indicating how they were calculated |

*Our web collection on statistics for biologists contains articles on many of the points above.*

## Software and code

Policy information about availability of computer code

| | |
|---|---|
| Data collection | FEI TEM user interface and serialEM 3.7.3 for EM images collection;<br>Bluice 5.0 for Crystallographic data collection;<br>Xcalibur 4.1 for Mass spectrometry data collection. |
| Data analysis | GraphPad Prism(version9), MotionCor2, CTFFIND4.1.8, relion3.1, cryoSPARC V2.5, EMAN2 command e2classsvsproj.py, HKL2000, CCP4 7.0, Xia2-dials, PHENIX-1.17.1, PyMol 2.3, PDBe PISA v1.52, HD-Examiner v2.3, BepiPred 2.0 web server, PatchDock v1.3 web server; |

For manuscripts utilizing custom algorithms or software that are central to the research but not yet described in published literature, software must be made available to editors and reviewers. We strongly encourage code deposition in a community repository (e.g. GitHub). See the Nature Portfolio guidelines for submitting code & software for further information.

## Data

Policy information about availability of data

All manuscripts must include a data availability statement. This statement should provide the following information, where applicable:

- Accession codes, unique identifiers, or web links for publicly available datasets
- A description of any restrictions on data availability
- For clinical datasets or third party data, please ensure that the statement adheres to our policy

Coordinates and structure factors have been deposited to the Protein Data Bank under accession codes 7VMZ, 7VN9 and 7VNG. All other data that support the

findings of this manuscript are available within the paper and its supplementary files. All other data are available from the corresponding authors upon reasonable request.

## Human research participants

Policy information about studies involving human research participants and Sex and Gender in Research.

| | |
|---|---|
| Reporting on sex and gender | None |
| Population characteristics | None |
| Recruitment | None |
| Ethics oversight | None |

Note that full information on the approval of the study protocol must also be provided in the manuscript.

# Field-specific reporting

Please select the one below that is the best fit for your research. If you are not sure, read the appropriate sections before making your selection.

☒ Life sciences ☐ Behavioural & social sciences ☐ Ecological, evolutionary & environmental sciences

For a reference copy of the document with all sections, see nature.com/documents/nr-reporting-summary-flat.pdf

# Life sciences study design

All studies must disclose on these points even when the disclosure is negative.

| | |
|---|---|
| Sample size | No sample size calculation was performed. For SPR experiments and pseudovirus neutralization assays, 3 biologically independent experiments were performed as indicated in related Figure legends and Methods section. For HDX-MS assays, continuous labeling hydrogen exchange experiments were independently repeated twice. |
| Data exclusions | No data was systematically excluded. The procedures of cryo-EM data analysis involves software ( relion3.1 & cryoSPARC V2.5) implemented sorting of particles that are damaged or are false-picked and are unlikely to refined correctly. During manual curation of Mass spectrometry data in HDExaminer, low quality peptides (peptides with low signal intensity) are removed. |
| Replication | Experimental findings were reliably reproduced. All experiments were successfully repeated at least twice (mostly three times) on separate occasions. |
| Randomization | Randomization was not relevant to our study. Because there's no allocation of samples/organisms/participants involved in our study. |
| Blinding | Blinding was not relevant to our study. Because there's no allocation of samples/organisms/participants involved in our study. |

# Reporting for specific materials, systems and methods

We require information from authors about some types of materials, experimental systems and methods used in many studies. Here, indicate whether each material, system or method listed is relevant to your study. If you are not sure if a list item applies to your research, read the appropriate section before selecting a response.

## Materials & experimental systems

| n/a | Involved in the study | |
|---|---|---|
| ☐ | ☒ | Antibodies |
| ☐ | ☒ | Eukaryotic cell lines |
| ☒ | ☐ | Palaeontology and archaeology |
| ☒ | ☐ | Animals and other organisms |
| ☒ | ☐ | Clinical data |
| ☒ | ☐ | Dual use research of concern |

## Methods

| n/a | Involved in the study | |
|---|---|---|
| ☒ | ☐ | ChIP-seq |
| ☒ | ☐ | Flow cytometry |
| ☒ | ☐ | MRI-based neuroimaging |

## Antibodies

| | |
|---|---|
| Antibodies used | In-house recombinant antibodies derived from synthetic phage display library were expressed as outlined in the Methods section. |
| Validation | Amounts of in-house antibodies were measured by nanodrop. The purity and homogeneity of in-house antibodies were analyzed by size-exclusion chromatography and coomassie blue stained SDS-PAGE gels. |

## Eukaryotic cell lines

Policy information about cell lines and Sex and Gender in Research

| | |
|---|---|
| Cell line source(s) | HEK293T cells, ATCC;<br>HEK293F cells, SF9 and High 5 cells, Thermo Fisher;<br>Caco2 cells, ATCC. |
| Authentication | Not authenticated after purchase. |
| Mycoplasma contamination | Cell lines have been tested negative for mycoplasma contamination by PCR methods. |
| Commonly misidentified lines<br>(See ICLAC register) | No commonly misidentified cell lines were used. |
